# Chemical Profiling and Evaluation of Antioxidant Activity of Artichoke (*Cynara cardunculus* var. *scolymus*) Leaf By-Products’ Extracts Obtained with Green Extraction Techniques

**DOI:** 10.3390/molecules29204816

**Published:** 2024-10-11

**Authors:** Valentina Masala, Stela Jokić, Krunoslav Aladić, Maja Molnar, Mattia Casula, Carlo Ignazio Giovanni Tuberoso

**Affiliations:** 1 Department of Life and Environmental Sciences, University of Cagliari, Cittadella Universitaria di Monserrato, S.P. Monserrato-Sestu km 0.700, 09042 Monserrato, Italy; valentina.masala2@unica.it (V.M.); mattia.casula@unica.it (M.C.); 2 Faculty of Food Technology Osijek, Josip Juraj Strossmayer University of Osijek, Franje Kuhača 18, 31000 Osijek, Croatia; kaladic@ptfos.hr (K.A.); mmolnar@ptfos.hr (M.M.)

**Keywords:** artichoke, leaves, by-products, green extraction, HPLC-PDA, LC–MS/MS, antioxidant activity, cynaropicrin

## Abstract

This study aimed to determine the effectiveness of different green extraction techniques (GETs) on targeted bioactive compounds from artichoke leaf by-products using deep eutectic solvent extraction (DESE), supercritical CO_2_ extraction (SCO_2_E), subcritical water extraction (SWE), and ultrasound-assisted extraction (UAE). Moreover, (HR) LC-ESI-QTOF MS/MS and HPLC-PDA analyses were used to perform qualitative–quantitative analysis on the extracts, enabling the detection of several bioactive compounds, including luteolin, luteolin 7-*O*-glucoside, luteolin 7-*O*-rutinoside, apigenin rutinoside, chlorogenic acid, and cynaropicrin as the most representative ones. SWE showed better results than the other GETs (TPC: 23.39 ± 1.87 mg/g of dry plant, dp) and appeared to be the best choice. Regarding UAE, the highest total phenols content (TPC) was obtained with 50:50% *v*/*v* ethanol: water (7.22 ± 0.58 mg/g dp). The DES obtained with choline chloride:levulinic acid showed the highest TPC (9.69 ± 0.87 mg/g dp). Meanwhile, SCO_2_E was a selective technique for the recovery of cynaropicrin (48.33 ± 2.42 mg/g dp). Furthermore, the study examined the antioxidant activity (1.10–8.82 mmol Fe^2+^/g dp and 3.37–31.12 mmol TEAC/g dp for DPPH^•^ and FRAP, respectively) and total phenols content via Folin–Ciocalteu’s assay (198.32–1433.32 mg GAE/g dp), of which the highest values were detected in the SWE extracts. The relationship among the GETs, antioxidant assays, and compounds detected was evaluated using Principal Component Analysis (PCA). PCA confirmed the strong antioxidant activity of SWE and showed comparable extraction yields for the antioxidant compounds between UAE and DESE. Consequently, GETs selection and extraction parameters optimization can be employed to enrich artichoke leaf by-products’ extracts with targeted bioactive compounds.

## 1. Introduction

Originating in the Mediterranean Basin, artichoke (*Cynara cardunculus* var. *scolymus*) is a diploid, mostly cross-pollinated species belonging to the Asteraceae family. It has been determined that the globe artichoke (var. *sativa* Moris, var. *scolymus* (L.) Fiori, ssp. *scolymus* (L.) Hegi) and the leafy or cultivated cardoon (var. *altilis* DC) are descended from the wild perennial taxon (var. *sylvestris* (Lamk) Fiori) [1]. Today, the primary globe-artichoke-growing zone is still the Mediterranean Basin. Italy is the world leader in the production of globe artichokes, with its coastal plains encompassing Sicily, Apulia, Sardinia, and Campania; Spain is the largest exporter, while France is an importer of both fresh and preserved artichokes [2]. The edible part of the globe artichoke is the inflorescence, the flower head forming at the top of the main stem and on the lateral shoots, composed of involucral bracts surrounding a fleshy base known as the heart, a natural source of minerals, fiber, inulin, and polyphenols, with very little fat content. It has been used as a medical plant, especially as a remedy for digestive problems, since the fourth century BCE, alone or in combination with other medicinal plants, such as *Curcuma longa*, *Achillea millefolium*, and *Gentiana lutea* [3].

Typically, approximately 30–40% of the total biomass is edible since only the flower base is edible in older buds. Furthermore, the amounts of the edible parts and waste are variable because they depend on the commercial use of the artichoke. For instance, fresh artichokes are sold by the piece in local markets, and the stem is cut at the insertion of the secondary buds [2,3]. For export or processing, it is cut shorter than 10 cm below the base of the bud. Finally, for other food uses, the flower bud is cleaned from the exterior leaves, and the inside of the artichoke is also cleaned. Currently, the leaves constitute the most interesting by-product. Globe artichoke leaf by-products have been used for several purposes: fertilizer or animal fodder [4,5], useful components regarding the nutritional, physical, and overall sensory quality of wheat bread [6], crackers [7], applications in cheese production due to their milk clotting and proteolytic activities [8,9], and natural antioxidant food additives [10].

*C. scolymus* leaves are rich in phenolic compounds, especially caffeoylquinic acid derivatives and flavonoids. Using the International Union of Pure and Applied Chemistry (IUPAC) nomenclature, based on the overall amount of caffeoylquinic acid, 5-*O*-caffeoylquinic acid (chlorogenic acid) is the most abundant single substance (39%), followed by 1,5-*O*-dicaffeoylquinic acid (21%) and 3,4-*O*-dicaffeoylquinic acid (11%) [4]. Among the flavonoids, the most representative are luteolin and apigenin and their derivatives, such as luteolin 7-*O*-glucoside (cynaroside), luteolin 7-*O*-rutinoside, apigenin 7-*O*-glucoside, and apigenin 7-*O*-rutinoside [11]. These compounds showed several biological activities. For instance, luteolin and cynaroside are involved in the inhibition of lipidic peroxidation and increase eNOS promoter activity and eNOS mRNA expression, whereas both caffeoylquinic acid derivatives and cynaroside demonstrate hepatoprotective activity against CCl_4_ toxicity in isolated rat hepatocytes [11].

Beyond flavonoids and caffeoylquinic acids, one of the most representative compounds is cynaropicrin, a sesquiterpene lactone of the guaianolide type. It is now considered to be a chemotaxonomic marker of artichoke plants [12] and is responsible for about 80% of the distinct bitter flavor of artichokes, which is linked to the activation of the bitter sensory receptors [13,14]. Cynaropicrin has gained interest in recent years for its several biological activities. Takei et al. [15] demonstrated that cynaropicrin has a possible use in protecting against photoaging and cosmetic problems due to its downregulation of the generation of ROS and the production of inflammatory cytokines including ultraviolet B-irradiated keratinocytes. It also demonstrates marked activity against anti-hepatitis C virus, anti-hyperlipidemic activity, anti-inflammatory activity, and anti-tumoral activity [16].

Several methods have been investigated in order to extract the bioactive components from *C. scolymus* leaves. Conventional methods such as Solid–Liquid Extraction (SLE) and UAE in ultrasonic bath [17,18], maceration in 75% *v*/*v* EtOH [11], and reflux conditions in methanol, heated to 70 °C for 1 h [18], have been used. UAE with a sonotrode is another innovative extraction technique that is a promising method for the recovery of bioactive compounds, enabling higher yields, short time, and low energy costs [19,20]. Furthermore, various green extraction techniques (GETs) such as pressurized hot water extraction, with water in its subcritical state [21,22], microwave-assisted extraction [23,24], and supercritical CO_2_ extraction [25] were performed. Subcritical water extraction (SWE) exploits water’s unique properties under these conditions, such as a lower dielectric constant, better diffusion, and a higher ionization constant, with a notable improvement in the phenol extraction yield when compared to the traditional extraction methods [26]. Moreover, supercritical CO_2_ extraction (SCO_2_E) was performed for the recovery of artichoke seed oil to transform it into biodiesel [27] and to extract oil and pentacyclic triterpenes from artichoke leaves and stalks [28]. One of the commonly investigated approaches to the extraction of bioactive compounds in the last decade is the use of deep eutectic solvents (DESs), alternative solvents employed for the development of a greener process, used, for instance, for the extraction of cynaropicrin [29]. Natural DESs (NaDESs) were also used to recover phenolic compounds from the outer petals of *C. scolymus* [30].

Taking into account the previous experiments performed so far, this study investigated the variations in the bioactive compounds and antioxidant activity of the obtained extracts from the *C. cardunculus* var. *scolymus* L. leaf by-products obtained with different GETs. It also aimed to evaluate the impact of these GETs on the aforementioned bioactive compounds. To achieve this, the following extraction techniques were carried out: DESE with choline chloride (ChCl) as a hydrogen bond acceptor (HBA) and various organic compounds as hydrogen bond donors (HBDs); UAE with a sonotrode at various amplitude and impulse values as well as H_2_O:EtOH ratios; SWE at various temperatures and H_2_O:EtOH ratios; and SCO_2_. Further research was conducted on UAE extracts using response surface methodology (RSM) and an applied Box–Behnken design (BBD). The bioactive components in the GET extracts from the artichoke leaves were investigated qualitatively and quantitatively using (HR) LC-ESI-QTOF MS/MS in the negative and positive ion modes and HPLC-PDA analysis. Additionally, the total polyphenol (TP) content determined via Folin–Ciocalteu’s assay and antioxidant activity (AA) determined by the DPPH^•^ (2,20-diphenyl-1-picrylhydrazyl radical), ABTS^•+^ (2,20-azinobis-(3-ethylbenzothiazoline-6-sulphonic acid)), FRAP (ferric reducing antioxidant power), and CUPRAC (cupric-ion-reducing antioxidant capacity) assays were assessed. Moreover, Principal Component Analysis (PCA) was used to estimate the association between the GETs, antioxidant assays, and targeted compounds.

## 2. Results and Discussion

*C. cardunculus* var. *scolymus* leaf by-products obtained from the commercialization of the edible floral bud with a portion of the stem (Figure S1) were extracted with four different GETs, and a total of seventeen samples were used for UAE, fourteen for SWE, sixteen for DESE, and one for SCO_2_E (Table 1). Based on the authors’ prior expertise, the parameters utilized for each GET were chosen [31,32,33,34].

### 2.1. Qualitative Determination of Bioactive Compounds in C. cardunculus var. scolymus Leaf Extracts

**Figure 1:**
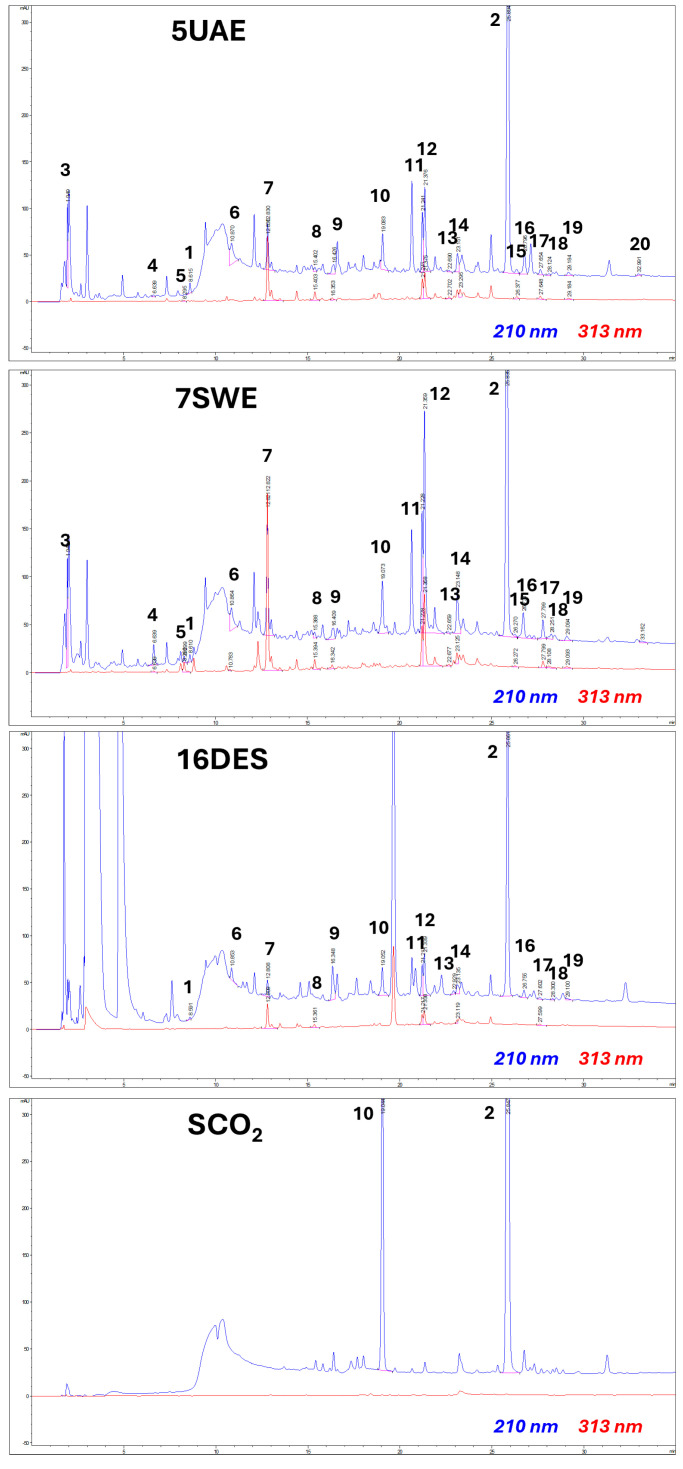
HPLC-PDA fingerprinting for selected *C. cardunculus* var. *scolymus* leaf by-product extracts (UAE: ultrasound-assisted extraction; SWE: subcritical water extraction; DES: deep eutectic solvents; SCO_2_: supercritical CO_2_ extraction) at λ = 210 and 313 nm. Peak identification is provided in Table S1. Chromatographic conditions are described in the text.

The forty-eight *C. cardunculus* var. *scolymus* leaf by-products’ extracts were qualitatively analyzed by (HR) LC-ESI-QTOF MS/MS in the negative and positive ion modes (Table S1), and 20 different targeted compounds were quantified by HPLC-PDA analysis (Figure 1). The compounds were identified by comparing the *m*/*z* values with those described in the literature and by comparing the experimental MS/MS spectra with the fragmentation patterns reported in the literature or with the fragmentation patterns and spectra reported in a public repository of mass spectral data [35,36]. Table S1 reports the compounds detected in the by-product extracts listed according to their LC-PDA retention times, along with the molecular formula derived by mass measurement (experimental result), MS/MS results, mass error (Δ ppm), the references used for identification, and the identification confidence levels [37]. Compounds **1** and **2** were detected in the positive ion mode and **3**–**20** were detected in the negative ion mode. Compound **1** was attributed to an amino acid, compound **3** to a cyclohexane carboxylic acid, and compound **16** to a furanoid lignan derivative. Compounds **2**, **9**, and **10** were attributed to sesquiterpene lactone derivatives, compounds **4** and **6** to hydroxybenzoic acid derivatives, compounds **5**, **7**, **8**, **13,** and **15** were attributed to hydroxycinnamic acid derivatives (mainly di-caffeoylquinic acid), and compounds **11**, **12**, **14**, and **17**–**20** were attributed to flavonoids (mainly luteolin and apigenin derivatives).

In detail, peak **1** was identified as tryptophan due to the [M + H]^+^ at *m*/*z* 205.0968 with fragments at 146.0599 and 118.0643 and due to the comparison with the pure standard and literature data [38]. Compound **2**, the tallest peak visible at 210 nm, was identified as cynaropicrin with the molecular formula C_19_H_22_O_6_ due to the [M + H]^+^ at *m*/*z* 364.1776 with an adduct with ammonia and a fragment at 181.1003 and the comparison with the literature data [39] and pure standard.

Compound **3** was identified as quinic acid due to the [M − H]^−^ at *m*/*z* 191.0558 and the comparison with the pure standard and literature data [40]. Compound **4** was identified as a protocatechuic acid derivative (hexoside) with molecular formula C_13_H_16_O_9_ due to the [M − H]^−^ at *m*/*z* 315.0718 with fragments at *m*/*z* 153.0175 (loss of a protocatechuic acid unit) and 152.0120; it was identified through the comparison with previous studies that showed the presence of protocatechuic acid derivatives in artichoke leaves [41]. Compound **5** was identified as neochlorogenic acid due to the [M − H]^−^ at *m*/*z* 353.0879 with fragments at *m*/*z* 191.0556 (loss of a quinic acid unit) and the comparison with the literature data [40,41,42]. Peak **6** was attributed to syringic acid hexoside due to the [M − H]^−^ at *m*/*z* 359.0982 with fragments at *m*/*z* 197.0456 and 182.0220 and the comparison with the literature data [42]. Compound **7** was attributed to chlorogenic acid due to [M − H]^−^ at *m*/*z* 353.0878 with fragments at *m*/*z* 191.0561 (loss of a quinic acid unit) and 179.0340. It was attributed due to the comparison with the pure standard and literature data [39,40,41,43]. Compound **8** was attributed to coumaroyl-quinic acid due to [M − H]^−^ at *m*/*z* 337.0936 with fragments at *m*/*z* 191.0562 (loss of a quinic acid unit) and 163.0391 and the comparison with previous studies [42]. Compound **9** was tentatively identified as cynaroscoloside C due to the [M − H]^−^ at *m*/*z* 471.1876 with fragments at *m*/*z* 59.0144 and 285.1841 and the comparison with previous studies [40]. Peak **10** was tentatively attributed to cynaroscoloside A/B due to the [M − H]^−^ at *m*/*z* 473.2035 with a fragment at 59.0139 and the comparison with the literature data [40]. Both compounds **9** and **10** form an adduct with formic acid. Peak **11** was attributed to luteolin 7-*O*-rutinoside due to the [M − H]^−^ at *m*/*z* 593.1524 with fragments at *m*/*z* 285.0405 (loss of a luteolin unit) and 284.0340. It was attributed also due to the comparison with the pure standard and literature data [11,39,41,42,43]. Compound **12** was identified as cynaroside (luteolin 7-*O*-glucoside) with the molecular formula C_21_H_20_O_11_. It is due to the [M − H]^−^ at *m*/*z* 447.0924 with fragments at 285.0396 (loss of a luteolin unit) and 284.0316 and the comparison with the literature data and pure standard [39,40,41,42,43]. Compound **13** was identified as di-caffeoylquinic acid I due to the [M − H]^−^ at *m*/*z* 515.1201 with fragments at *m*/*z* 191.0563 (loss of a quinic acid unit), 353.0779 (loss of a caffeoylquinic acid unit), and 135.0452, and the comparison with previous studies [11,39,41,42,43]. Peak **14** was attributed to apigenin rutinoside due to the [M − H]^−^ at *m*/*z* 577.1576 with a fragment at *m*/*z* 269.0428 (loss of an apigenin unit) and with the comparison with the literature data [11,41,42].

Compound **15** was tentatively identified as a di-caffeoylquinic acid II with the molecular formula C_25_H2_24_O_12_ due to the [M − H]^−^ at *m*/*z* 561.1622 (with an adduct with formic acid) with fragments at 515.1644 and 191.0554 (loss of a quinic acid unit) and the comparison with the literature data [11,39,41,42,43]. Compound **16** was tentatively identified as pinoresinol hexoside with the molecular formula C_26_H_31_O_11_ due to the [M − H]^−^ at *m*/*z* 519.1864 with a fragment at *m*/*z* 151.0385 and 357.1323 and the comparison with the literature data [42,43]. Compound **17** was tentatively identified as apigenin glucuronide with the molecular formula C_21_H_18_O_11_ due to the [M − H]^−^ at *m*/*z* 445.0778 with a fragment at *m*/*z* 269.0448 (loss of an apigenin unit) and the comparison with the literature data [42]. Peak **18** was attributed to luteolin glucoside with an acetyl moiety with the molecular formula C_23_H_22_O_12_ due to the [M − H]^−^ at *m*/*z* 489.1044 with fragments at *m*/*z* 285.0397 (loss of a luteolin unit) and 284.0307 and the comparison with the literature data [40,43]. Compound **19** was tentatively identified as luteolin aglycone due to the [M − H]^−^ at *m*/*z* 285.0797 and the comparison with the pure standard and with previous studies [40,41,42,43]. Peak **20** was attributed to apigenin aglycone due to the [M − H]^−^ at *m*/*z* 269.0456 and the comparison with the pure standard and literature data [42].

*C. cardunculus* var. *scolymus* leaf by-products’ extracts showed substantial similarity with the literature data, confirming that the most representative compounds are caffeoylquinic acid derivatives, flavonoids, and cynaropicrin [4,11,12]. Interestingly, UAE and SWE showed similar and comparable chromatograms. Regarding DESE, the chromatogram was very similar to that of UAE and SWE, but with differences and limitations in the signal regarding flavonoids and caffeoylquinic acids, while cynaropicrin remains equally expressed. Cynaroscoloside A/B and cynaropicrin were the two very representative peaks that SCO_2_E displayed. The other classes of compounds were limited, as indicated by the total absence of any notable peaks.

### 2.2. Quantitative Determination of Bioactive Compounds in C. cardunculus var. scolymus Leaf Extracts and Influence of Extraction Technique on Selected Compounds Content

**Figure 2:**
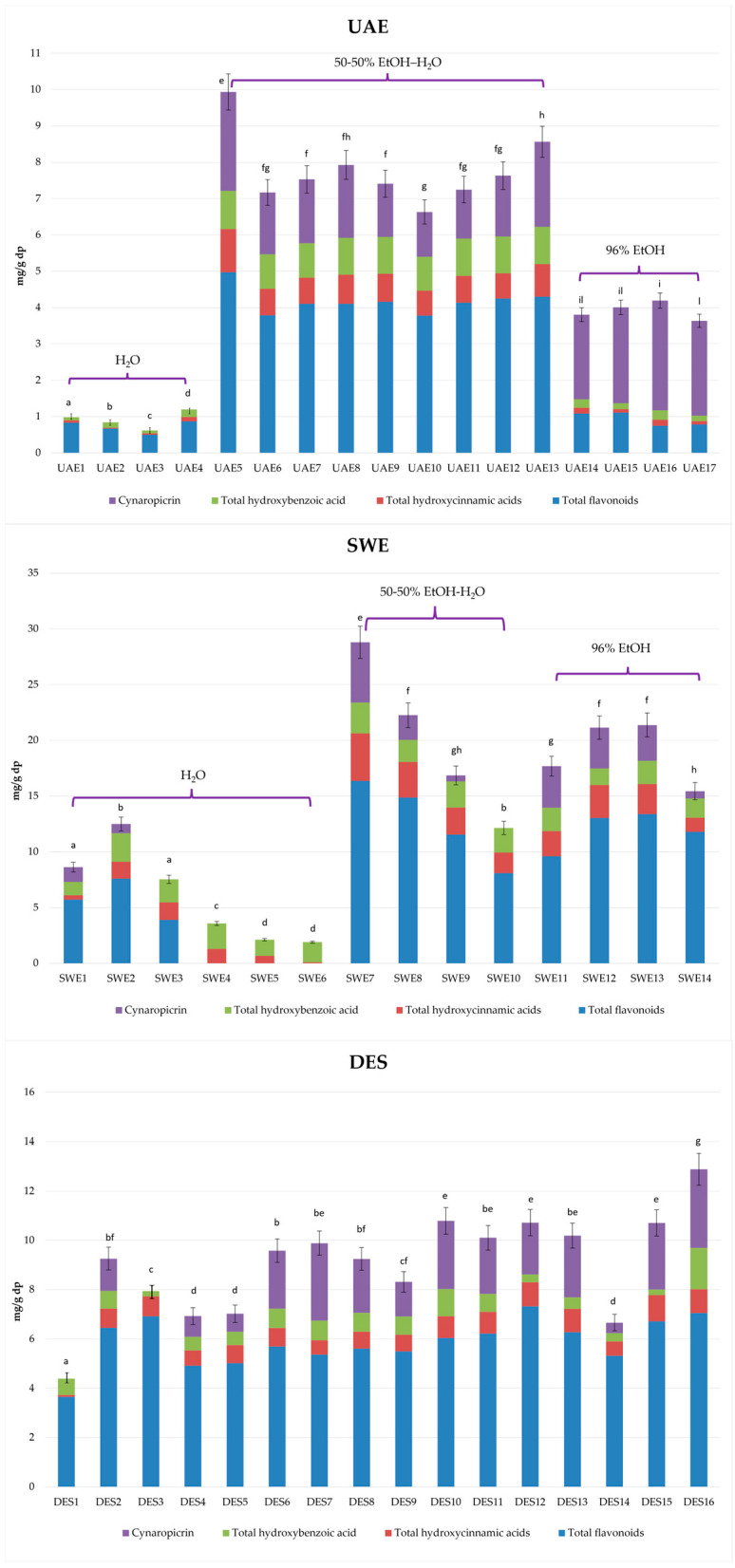
Quantification of phenolic compounds by LC-PDA method (mg/g dp) in *C. cardunculus* var. *scolymus* leaf by-products’ extracts. Data are provided as mean ± standard deviation (*n* = 3). Mean values with different letters are significantly different (homogenous groups) at *p* ≤ 0.05. Table S2 reports full dataset (mean ± standard deviation; *n* = 3) and the ANOVA test to determine the statistically significant differences among homogenous groups.

Figure 2 shows the total amount of hydroxycinnamic acid derivatives, flavonoids, hydroxybenzoic acids, and cynaropicrin in all the types of extracts, and Table S2a–c report the quantification of the target compounds by the LC-PDA method (amount expressed as mg/g of dry plant, dp). The comparison of the data obtained from the four different GETs highlighted how they can influence the extraction and how they can be selective for a specific class or single compounds.

Taking into account the most abundant compounds, UAE, SWE, and DESE showed average values of 27.63%, 34.71%, and 25.34% for luteolin-7-*O*-glucoside (the most abundant among the flavonoids), 40.49%, 56.37%, and 63.04%, respectively, for chlorogenic acid (the most abundant among the hydroxycinnamic acids), and 63.42%, 45.85%, and 71.43% for cynaropicrin. SCO_2_E enabled the extraction of an average value of 51.23% of luteolin-7-*O*-glucoside, and, regarding the other compounds, the only representative one was cynaropicrin.

#### 2.2.1. Ultrasound-Assisted Extraction (UAE) with Sonotrode

Extracts with 100% H_2_O, EtOH:H_2_O (50:50, *v*/*v*), and 96% EtOH as solvents were set at different values of the chosen process parameters according to the response surface methodology (RSM) and applied Box–Behnken design (BBD) [44]. The amounts of the chosen phenolic components in the UAE extracts varied depending on the extraction conditions used (Table S2a). Among the UAE extracts, run 5UAE (EtOH:H_2_O (50:50, *v*/*v*) and both amplitude and impulse set at 100) is the one with the highest total phenols content (TPC) (7.22 ± 0.58 mg/g dp) but also with the highest total flavonoids content (TFC) (4.98 ± 0.40 mg/g dp), total hydroxycinnamic acids content (THC) (1.18 ± 0.06 mg/g dp), and total hydroxybenzoic acid content (THB) (1.06 ± 0.09 mg/g dp), higher than the extracts created with the other two solvents but on par with the extracts created with the same solvent (runs 6UAE, 7UAE, 8UAE, 9UAE, 11UAE, 12UAE, and 13UAE).

It is interesting that the highest amount of cynaropicrin is represented by run 16UAE (3.02 ± 0.29 mg/g dp) with 96% EtOH as the solvent (amplitude 60 and impulse 100). The lowest amount in phenols is represented by the extracts obtained with 100% water (1UAE, 2UAE, 3UAE, and 4UAE), in particular 3UAE with a TPC of 0.62 ± 0.03 mg/g dp, a TFC of 0.50 ± 0.05 mg/g dp, a THB of 0.08 ± 0.01 mg/g dp, and cynaropicrin was not detected. Instead, the lowest THC was represented by run 2UAE (0.02 ± 0.00 mg/g dp).

**Figure 3:**
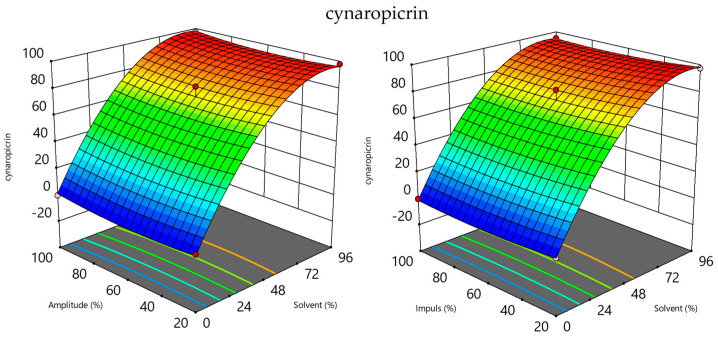
Three-dimensional plots for obtained cynaropicrin in extracts as a function of UAE process parameters.

The most crucial UAE operational parameters (solvent type, amplitude, and impulse) were optimized using BBD in order to produce the greatest concentration of the most prevalent compounds found (luteolin 7-*O*-rutinoside, luteolin 7-*O*-glucoside, apigenin rutinoside, luteolin, chlorogenic acid, and cynaropicrin). Table S4 provides the coefficients and the corresponding *p*-values for each response under investigation in the experiments. Multiple linear regression analysis was utilized to ascertain the regression coefficients. The *p*-value indicates each factor’s level of statistical significance. It is clear from the results that the type of solvent used can significantly affect the extraction performance (linear or quadratic terms) and, ultimately, the extracts that contain the targeted compounds (Figure S2a–e and Figure 3). The solvent’s quadratic term had the greatest statistically significant impact on the following responses under investigation: luteolin 7-*O*-rutinoside, apigenin rutinoside, luteolin, chlorogenic acid, and cynaropicrin, while the linear term had a significant influence on luteolin 7-*O*-glucoside as well as on luteolin, chlorogenic acid, and cynaropicrin.

This is also evident from the 3D graphs for the target compounds, where, for example, in Figure 3, the strong influence of the solvent type on the cynaropicrin content can be observed. With the increase in the ethanol concentration, the cynaropicrin content in the obtained extracts also significantly increased. The UAE process, which involves the isolation of bioactive compounds, is influenced by various parameters like extraction time, ultrasonic power, and solvent concentration. The extraction solvent, such as EtOH and EtOH:H_2_O mixtures, is crucial for phenolic extraction. Pure ethanol reduces the extraction efficiency due to the hydrophilic nature of phenols, while pure ethanol positively affects cynaropicrin, a weakly polar compound. When choosing the solvent, selectivity, safety, cost, and availability must be taken into account.

By reviewing the literature, Bràs et al. [45] studied the effect of the pulse mode solid/liquid ratio, amplitude, and temperature upon cynaropicrin extraction from *C. cardunculus* leaves. They optimized the extraction to obtain 192.51 ± 6.96 mg/g of the dry weight, with an extraction yield of 23.90 ± 0.14 mg/g of the dry weight with a solid/liquid ratio of 1/35, amplitude 50%, time 30 min, and temperature set at 45 °C. Although the extraction conditions were different and not comparable to those used in this study, this can confirm UAE as a promising method for the extraction of selected compounds. Moreover, Saleh et al. [20] investigated the chlorogenic acid yield by using both UAE with a probe and with a sonic bath and 80% methanol as the solvent. The highest yield was represented by UAE using a 20 kHz probe after 15 min of extraction, followed by UAE with a 40 kHz water bath for 60 min, confirming that UAE with a probe is a better extraction technique than the conventional methods, such as maceration, boiling, and the use of Soxhlet extraction with 80% methanol.

#### 2.2.2. Supercritical CO_2_ Extraction (SCO_2_E)

For SCO_2_E, only one set of process conditions were performed: a temperature of 40 °C and pressure of 300 bar to observe the selectivity of this process. The SCO_2_ extract showed the highest amounts of cynaropicrin (48.33 ± 2.42 mg/g dp) and cynaroscoloside A/B (8.22 ± 0.74 mg/g dp) (Table S2a), both sesquiterpene lactones. Interestingly, it showed a TPC of only 1.53 ± 0.14 mg/g dp, in which it was possible to identify and quantify only luteolin 7-*O*-rutinoside and luteolin 7-*O*-glucoside. The amount of THC was considerably low (0.08 ± 0.01 mg/g dp), whereas hydroxybenzoic acids were not detected at all. These results are consistent with the restricted capacity of SCO_2_E to extract polar compounds, while it is focused on non-polar compounds [46]. In this way, SCO_2_E has several advantages: it is an environmentally friendly process and produces solvent-free extracts, it has a minimal alteration of the bioactive compounds, and it can be highly selective in terms of extraction. Notably, supercritical fluid extraction that is properly modified has been previously used to remove the bitterness from *Leuzea carthamoides* leaves [47]. SCO_2_E is a versatile extraction method that can be adjusted by changing the temperature or pressure to alter the CO_2_ density, thus regulating the solubility. SCO_2_ is primarily used for extracting high-value chemicals from non-polar to mid-polar molecules like essential oils. Modifiers, or cosolvents, can be added to increase the extraction efficiency due to SCO_2_E’s limited capacity to extract highly polar molecules. Understanding the thermodynamics and kinetics of SCO_2_E is crucial for ensuring high extraction selectivity and minimizing non-target co-extraction [46]. Since cynaropicrin is a weakly polar molecule, it is possible to extract it with this extraction technique. In Figure 1, the SCO_2_ chromatogram supports the selectivity of the method for non-polar compounds, pointing out two peaks corresponding to cynaroscoloside A/B and cynaropicrin. Therefore, this GET could have a possible future use to isolate the molecules of our interest. For instance, it could be applied regarding cynaropicrin,, a compound with strong biological activities [12,13,14,15,16], such as antiphotoaging activity, by inhibiting the NF-KB-mediated transactivation in mouse models [48], in vitro anti-inflammatory effects [49], and in vivo activity against *Trypanosoma brucei* [50], and it causes the potent inhibition of hematopoietic tumoral cells in vitro and in vivo in multiple myeloma [51].

#### 2.2.3. Subcritical Water Extraction (SWE)

The SWE extraction was performed at six different growing temperatures using 100% water and with four increasing temperatures using EtOH:H_2_O (50:50, *v*/*v*) and 96% EtOH as the solvents (Table 1). All the SWE extracts showed a trend that is globally greater than that of the other extraction techniques (Table S2b), except for flavonoids, which, extracted with 100% water, are subject to degradation as the temperatures increase [52]; due to this, they have not been detected (4SWE, 5SWE, and 6SWE). Among the extracts obtained with 100% water, the highest TPC is represented by 2SWE (150 °C) (11.68 ± 1.05 mg/g dp). Interestingly, 7SWE (EtOH:H_2_O (50:50, *v*/*v*), 125 °C) showed the highest amounts of TPC, TFC, THC, and THB among all the extracts obtained with the selected GETs (23.39 ± 1.87 mg/g dp, 16.36 ± 1.47 mg/g dp, 4.26 ± 0.30 mg/g dp and 2.77 ± 0.25 mg/g dp, respectively). Moreover, 7SWE demonstrated the highest amounts of luteolin 7-*O*-glucoside, luteolin 7-*O*-rutinoside, chlorogenic acid, and cynaropicrin (7.50 ± 0.38 mg/g dp, 3.49 ± 0.33 mg/g dp, 3.28 ± 0.20 mg/g dp and 5.41 ± 0.43 mg/g dp, respectively), while it indicated one of the lowest amounts of protocatechuic acid hexoside (0.16 ± 0.02 mg/g dp). Notably, 3SWE (175 °C, 100% water) showed the highest amount of protocatechuic acid hexoside (0.92 ± 0.09 mg/g dp), and 10SWE (200 °C, EtOH:H_2_O (50:50, *v*/*v*)) displayed the highest amount of luteolin aglycone (4.20 ± 0.33 mg/g dp). Between the extracts obtained with 96% EtOH, 12SWE and 13SWE showed the highest amounts of TFC, THC, and TPC (13.04 ± 1.04 mg/g dp and 13.38 ± 0.80 mg/g dp, 2.96 ± 0.27 mg/g dp and 2.70 ± 0.24 mg/g dp, 17.48 ± 1.25 mg/g dp and 18.16 ± 1.63 mg/g dp), while the highest amount of THB was represented by 11SWE and 13SWE (2.12 ± 0.17 mg/g dp and 2.08 ± 0.17 mg/g dp). Concerning EtOH:H_2_O (50:50, *v*/*v*), from run 7SWE to run 10SWE, it demonstrated a marked decrease in the amounts of all the identified compounds linked to the rise in temperature. Among the extracts in which the selected compounds were detected, the lowest amounts of luteolin 7-*O*-glucoside and luteolin 7-*O*-rutinoside were demonstrated in run 10SWE (1.18 ± 0.10 mg/g dp and 1.04 ± 0.08 mg/g dp), the lowest amount of chlorogenic acid was obtained in run 1SWE (0.08 ± 0.01 mg/g dp), the lowest amount of protocatechuic acid hexoside was determined in 11SWE (0.09 ± 0.00 mg/g dp), and the lowest amount of cynaropicrin was shown in run 3SWE and run 4SWE, where it was detected in traces, and, in runs 5SWE, 6SWE, and 10SWE, it was completely absent. To the best of our knowledge, there are no studies that describe the same method with the same parameters, especially using EtOH:H_2_O (50:50, *v*/*v*) and 96% EtOH. Órbenes et al. [22] used water in its subcritical state in a temperature range of 140–240 °C. This study showed maximum TPC values of 2.9 and 3.8 g GAE/100 mg regarding artichoke leaves at 220 °C.

#### 2.2.4. Deep Eutectic Solvent Extraction (DESE)

The DES extracts represent a heterogeneous group of samples, and all 16 of the investigated solvents exhibited comparable behaviors (Table S2c). The one with the highest TPC and THB is 16DES, with amounts of 9.69 ± 0.87 mg/g dp and 1.67 ± 0.13 mg/g dp, respectively. Interestingly, DESs globally showed the absence of protocatechuic acid hexoside, except for 2DES with a small amount of 0.05 ± 0.01 mg/g dp. The lowest amount of THB was obtained by 15DES (0.23 ± 0.01 mg/g dp), which, on the other hand, showed the highest amounts of THC (1.07 ± 0.08 mg/g dp) and chlorogenic acid (0.68 ± 0.07 mg/g dp). 12DES showed the highest amount of TFC (7.33 ± 0.59 mg/g dp), while 1DES showed the lowest (3.65 ± 0.33 mg/g dp). Luteolin 7-*O*-glucoside and luteolin 7-*O*-rutinoside showed the highest amounts in 12DES (2.30 ± 0.21 mg/g dp and 1.28 ± 0.06 mg/g dp, respectively), in line with the highest TFC and the lowest in 1DES (0.71 ± 0.04 mg/g dp and 0.55 ± 0.04 mg/g dp, respectively). Luteolin aglycone was not detected in 1DES, and it was approximately constant in all the extracts. Cynaroscoloside A/B was found in traces in all 16 extracts, while cynaropicrin was not detected in 3DES, but the highest amounts were found in 7DES and 16DES (3.14 ± 0.25 mg/g dp and 3.19 ± 0.26 mg/g dp, respectively), two different solvents (choline chloride: butane-1,4-diol and choline chloride:levulinic acid, respectively) but with a comparable amount of cynaropicrin. Although there are no previous studies that used the same DES extraction techniques, De Faria et al. [29] used different DESs for the recovery of cynaropicrin. The highest extraction yield (2.84% total weight) was obtained with a DES mixture of decanoic acid:[N_4444_]Cl (2:1), probably due to a decrease in the viscosity of the solvent. Notably, there is an increase in the cynaropicrin extraction yield by decreasing the amount of the HBA species. However, in our study, the highest amount of cynaropicrin was found in 16DES (3.19 ± 0.26 mg/g dp) composed of choline chloride:levulinic acid 1:2.

### 2.3. Antioxidant Activity and Total Phenols in C. cardunculus var. scolymus Leaf Extracts

Five spectrophotometric methods were selected to assess the antioxidant activity and total phenolic compound quantification in the *C. cardunculus* var. *scolymus* leaf by-products’ extracts (Table 2). Namely, two total antioxidant capacity assays (CUPRAC and FRAP), two free-radical-scavenging activity (DPPH^•^ and ABTS^•+^) assays, and the Folin–Ciocalteu assay that is based on a redox reaction [53] were used.

Overall, the highest antioxidant activity (AA) values (1.10–8.82 mmol TEAC/g dp and 3.37–31.12 mmol Fe^2+^/g dp, for DPPH^•^ and FRAP, respectively) and TP values (198.32–1433.32 mg GAE/g dp) were detected in the SWE extracts. Regarding TP and other antioxidant and antiradical assays, sample 3DES showed an unusual color reaction, resulting in abnormal values. For this reason, these data were not included in the discussion (not measurable, Table 2). Decreases in AA and TP were observed at the lowest temperatures (125 °C). In addition, the TP and AA perspectives indicated that the most valuable extraction circumstances were 10SWE (200 °C, EtOH: H_2_O (50:50, *v*/*v*)). Moreover, the extracts created with UAE showed significant levels of AA (0.17–1.23 mmol TEAC/g dm, 0.31–3.07, and 0.05–5.44 mmol Fe^2+^/g dm, for ABTS^•+^, FRAP, and CUPRAC, respectively) and were rich in polyphenols (247.62 ± 17.53 and 289.33 ± 60.78 mg GAE/g dm, for 1UAE and 4UAE, respectively). The DES extracts showed moderate AA and TP values and almost constant values; the TP values for these extracts ranged from 16.63 to 134.45 mg GAE/g dp, and the AA values ranged from 0.01 to 2.83 mmol Fe^2+^/g dp and from 0.19 to 1.33 mmol TEAC/g dp (DPPH^•^ and ABTS^•+^, respectively) and were 0.18–4.25 and 0.37–2.36 mmol Fe^2+^/g dp (FRAP and CUPRAC, respectively).

All the spectrophotometric assays thus performed showed a highly significant positive correlation (R^2^ ≥ 0.9454, *p* ≤ 0.001) with each other (Table S3). Pearson’s correlation coefficients among the antioxidant assays and the bioactive compounds highlighted significant positive correlations (*p* ≤ 0.05, *p* ≤ 0.01, and *p* ≤ 0.001) with several compounds (Table S3). Regarding the selected classes of compounds, the one with the highest AA was the hydroxybenzoic acids due to the highest AA of protocatechuic acid hexoside. Among flavonoids, luteolin aglycone is the one with the most optimal Pearson’s correlation coefficients and the most optimal AA. Similarly, pinoresinol hexoside had the highest AA among the other compounds (*p* ≤ 0.001), followed by hydroxycinnamic acids, apigenin aglycone, other flavonoids, and dicaffeoylquinic acid I.

**Figure 4:**
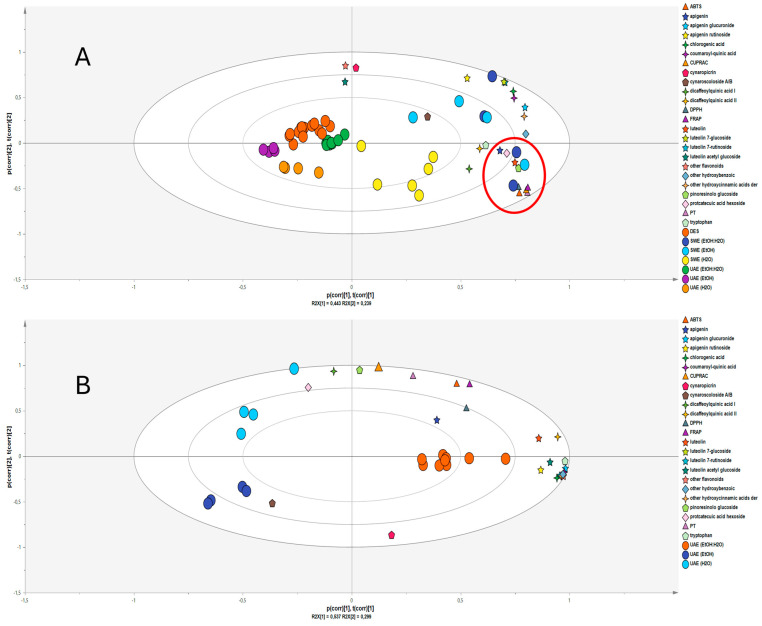
Principal Component Analysis (PCA) score scatter biplot of the extracts obtained from *C. cardunculus* var. *scolymus* samples by using (**A**) ultrasound-assisted extraction (UAE), subcritical water extraction (SWE), and deep eutectic solvents (DESs; *n* = 46) and (**B**) ultrasound-assisted extraction (UAE) (*n* = 17).

Principal Component Analysis (PCA) was performed to further investigate the relationships among the extraction techniques, antioxidant assays, and classes or single compounds detected in the extracts. A biplot approach (Figure 4A) was taken to better emphasize the similarities or differences among the groups. The samples obtained using DESs appear to be clustered together on the left side of the PCA biplot. This suggests that these samples may share similar characteristics in terms of their antioxidant properties and the compounds detected. The UAE groups showed three distinct clusters within the group and some overlap with the DES samples. In particular, the UAE group (EtOH: H_2_O (50:50, *v*/*v*)) appeared to be located close to the DES group. This suggests that the two techniques might provide similar extraction yields. The SWE samples showed a high spread and two distinct clusters within the group.

All the antioxidant assays and TP are located close to the samples obtained by using SWE with 96% ethanol and EtOH:H_2_O (50:50, *v*/*v*) as the extraction solvents, and they appear to overlap, confirming the results of Pearson’s correlation (Table S3). Furthermore, this antioxidant activity may be linked to specific metabolites such as luteolin, pinoresinol hexoside, apigenin, protocatechuic acid hexoside, other hydroxybenzoic acids, and other hydroxycinnamic acids, as shown by the results of Pearson’s correlation. Interestingly, cynaropicrin, despite being one of the most present compounds, is not correlated with antioxidant activity.

To further investigate the differences underlined by Figure 4A within the samples group obtained by using UAE, a new PCA was performed (Figure 4B). As already observed in Figure 4A, the UAE samples were spread and formed three distinct clusters within the group. Furthermore, while the clusters UAE (96% EtOH) and UAE (100% H_2_O) have shown a weak correlation with the metabolites, UAE (EtOH:H_2_O (50:50, *v*/*v*)) appeared to be highly correlated with most of the metabolites. This result suggests that, among the three methods based on UAE, using a mixture of ethanol/water coupled with ultrasound may be the better compromise to obtain a high yield of antioxidant compounds.

By reviewing the literature, several studies evaluated the AA and TP values of artichoke by-products. Peschel et al. [54] evaluated the TP and AA in eleven fruit and vegetable by-products, artichoke included, extracted with conventional methods. It is interesting to note that they confirm that ethanol as a solvent has a higher TP value than water alone (88.15 ± 4.99 and 42.75 ± 12.17 mg GAE/g of dry extract, respectively). Interestingly, it has been studied that artichoke by-products have a higher TP value than the heart (14.16 ± 0.08 and 9.06 ± 0.06 mg GAE/g dry weight, respectively) and also a higher total flavonoids content (TFC) (9.85 ± 0.12 and 5.91 ± 0.12 mg quercetin equivalents/g fresh weight) [55], and this is intriguing in light of the good results thus obtained. Furthermore, Llorach et al. [56] demonstrated that the conventional extracts from artichoke by-products have strong scavenging activity against both DPPH^•^ and ABTS^•+^ radicals. They highlighted that the highest antiradical activity versus DPPH^•^ was found in methanol extracts, probably because of non-polar compounds with antioxidant capacity, mainly fiber. In this regard, focusing on SWE, the highest antiradical activity versus DPPH^•^ was in 11SWE (8.82 ± 0.56 mmol Fe^2+^/g dp), extracted with EtOH: H_2_O (50:50, *v*/*v*), and the lowest was in 1SWE (1.10 ± 0.01 mmol Fe^2+^/g dp), extracted with 100% water.

Concentrating on individual compounds, protocatechuic acid hexoside is one of the compounds with the highest antioxidant activity. Protocatechuic acid has been studied before for its antioxidant activity, especially in vitro using DPPH^•^, ABTS^•+^, FRAP, and CUPRAC [57]. Pinoresinol hexoside is another compound with potent antioxidant properties, which can be linked to the fact that pinoresinol-4-*O*-*β*-D-glucopyranoside from *Prunus domestica* showed promising antioxidant activity by using FRAP and ABTS^•+^ analyses (418.47 and 1091.3 µmol/g in terms of ascorbic acid, respectively) [58]. Additionally, the antioxidant activity and radical scavenging activities of luteolin and apigenin were confirmed by DPPH^•^, ABTS^•+^, and FRAP analyses in previous studies [59].

## 3. Materials and Methods

### 3.1. Chemicals

All the chemicals were of analytical grade. The solvents used for the extraction were purchased from J.T. Baker (Radnor, PA, USA). Methanol and 85% *w*/*w* phosphoric acid were purchased from Sigma-Aldrich (Steinheim, Germany). LC–MS-grade acetonitrile, formic acid, and H_2_O were purchased from Merck (Darmastadt, Germany). Standard flavonoids were purchased from Extrasynthese (Genay Cedex, France) and TransMIT (Giessen, Germany). The Folin–Ciocalteu reagent together with 2,2-diphenyl1-picrylhydrazyl radical (DPPH^•^) and gallic acid were purchased from Sigma-Aldrich (St. Louis, MO, USA). Ferrous sulphate, copper(II) chloride dihydrate, ammonium acetate, neocuproine (2,9-dimethyl-1,10-phenanthroline) hydrochloride, (±)-6-hydroxy-2,5,7,8-tetramethylchroman-2-carboxylic acid (Trolox), 2,20-azino-bis(3-ethylbenzothiazoline-6-sulfonate radical cation (ABTS^•+^), potassium persulphate, acetic acid, ferric chloride, copper(II) sulphate pentahydrate, 2,4,6-tris(2-pyridyl)-1,3,5-triazine (TPTZ), and sodium acetate trihydrate were obtained from Merck-Sigma-Aldrich (Milan, Italy). Ultrapure water (18 MΩ·cm) was obtained with a Milli-Q Advantage A10 System (Millipore, Milan, Italy).

### 3.2. Plant Material

*C. cardunculus scolymus* (cv. Imperial star, Galassi sementi Gambettola, FC, Italy) leaf by-products were collected in January 2023 in Assemini (Sardinia, Italy). These leaves represent those that are generally discarded before the commercialization of the fresh artichoke, and they appeared to be the largest and most external leaves of the plant. After the collection, the leaves were gently cleaned and dried at 45 °C for 24 h (Hendi Dehydrator Profi Line, De Klomp, The Netherlands). Before extraction procedures, the dried leaves were homogenized and ground using a standard laboratory mill to obtain a powder sample (Figure S1). The dry plant residue (dp) was evaluated in triplicate by drying 10 g of by-product for 5 h in a thermostatic oven at 105 ± 1 °C to a constant weight.

### 3.3. Extraction Techniques

UAE, SWE, DESE, and SFE have been previously described [33,34]. For UAE, powdered samples were placed in different solvents and extracted with an ultrasonic probe (UP400St, Hielscher Utrasonics GmbH, Teltow, Germany) set at a minimum power of 400 W and a frequency of 24 kHz. RSM and Box–Behnken design were applied (changing solvent type, amplitude, and impulse, Table 1), and data analysis was performed using Design-Expert software^®^ ver.9 (Stat-Ease Inc., Minneapolis, MN, USA) and ANOVA [34] to evaluate the quality of the fitted model. The SWE process was performed by placing the powdered sample in a stainless-steel vessel with the proper solvents, heating it at various temperatures (Table 1), stirring it with a magnetic stirrer, and using N_2_ to control pressure. The reactor content was then cooled in an ice bath and filtered through filter paper to obtain the extracts. For DES extraction, the method reported in a previously published paper by Masala et al. [34] was used. DES mixtures were prepared using different HBD/HBA ratios, with 16 different HBDs chosen based on molecular weight and ratio (Table 1). Glass beads were weighed in vials and filled with solvents and H_2_O Milli Q. Sample analyses were performed in a Bead Ruptor 12 (Omni International, Inc., Kennesaw, GA, USA) in triplicate, homogenized, and centrifuged before being collected into 1 mL Eppendorf tubes. Regarding Supercritical CO_2_ extraction, it was performed in the Supercritical Fluids Extraction system with a properly modified process, previously described [33,60]. Briefly, the powdered samples (125 g) were placed in an extractor vessel, and the extracts were collected in previously weighted glass tubes at 15 bar and 25 °C. The extractions were performed at a temperature of 40 °C for 60 min at 300 bar, and the CO_2_ mass flow rate was 1.4 kg CO_2_/h.

### 3.4. High-Resolution HPLC-ESI-QToF-MS/MS and HPLC-DAD Analyses

The method outlined by De Luca et al. [61] was applied for the qualitative and quantitative evaluation of the artichoke by-product. An electrospray ionization (ESI) source configured to operate in both positive and negative ion modes was used for the experiments. In summary, the analytical setup consisted of an advanced ion mobility QToF LC/MS system with a 1290 Infinity II UPLC and a 6560 IM-QToF (Agilent Technologies Inc., Palo Alto, CA, USA). The MassHunter Workstation Qualitative Analysis software version 10.0 (Agilent Technologies) was then used to evaluate the ESI/QToF MS data. The metabolites were tentatively identified using the MassHunter METLIN metabolite PCDL database v. B.08.00 (Agilent Technologies) and the Sirius^®^ software v. 4.7.4 to predict fragmentation and molecular formulae [35,62]. Additionally, the experimental MS/MS spectra were compared with fragmentation patterns published in the literature or with spectra published in a publicly accessible mass spectral data repository [36]. Using an Agilent Technologies G4212B photodiode array detector, a 1260 Infinity II HPLC system was utilized to conduct a quantitative analysis of phenolic compounds. OpenLab CDS software v. 2.5 (Agilent Technologies) was used to process the chromatograms and spectra. Phenolic compounds were identified and quantified by measuring their absorption at characteristic wavelengths (flavonols at 360 nm, hydroxycinnamic acids at 313 nm, hydroxybenzoic acids at 280 nm, tryptophan, and sesquiterpenes at 210 nm).

Using the least squares approach to correlate the peak area with the concentration, the calibration curves were built, with R^2^ > 0.999 for all standards in the 0.2–10.0 mg/L range. For the analysis, the extracts were diluted 1:10 *v*/*v* for the UAE, DES, and SCO_2_ samples and 1:20 for the SWE samples using 0.22 M phosphoric acid. Prior to injection, the solutions were filtered using a 0.22 μm CA syringe filter.

### 3.5. Determination of Total Polyphenol Content (Folin–Ciocalteu’s Assay), Free Radical Scavenging Activity (ABTS^•+^ and DPPH^•^ Assays), and Total Reducing Power (CUPRAC and FRAP Assays)

Using 10 mm Kartell^®^ plastic cuvettes, all tests were performed on a Cary 50 spectrophotometer (Varian, Leinì, TO, Italy). In order to suit the calibration curve ranges, samples were appropriately diluted with MeOH in the 1:1–1:100 *v*/*v* range before the analysis. The modified Folin–Ciocalteu spectrophotometric method was used to assess the total polyphenol (TP) content [63,64].

In short, 500 µL of Folin–Ciocalteu reagent was combined with 100 µL of the diluted sample, and, after 5 min, 3 mL of 10% Na_2_CO_3_ (*w*/*v*) was added. After agitating the mixture and diluting it with water to a final volume of 10 mL, it was allowed to sit at room temperature for 90 min. At 725 nm, the absorbance was measured in relation to a blank. Using a calibration curve of a recently prepared gallic acid standard solution (10–200 mg/L), the TPC results were reported as mg of gallic acid equivalent (GAE) per g of residue.

The DPPH^•^ was carried out in accordance with Tuberoso et al. [63]. Two mL of DPPH^•^ solution (0.04 mmol/L in methanol) was applied to 10 mm cuvettes along with 50 µL of diluted extract or standard. After 60 min, the spectrophotometric measurements were taken at 517 nm. The ABTS^•+^ tests were carried out in accordance with Re et al. 1999 [65] with some modifications [64]. The ABTS stock solution was reacted with 70 mM potassium persulfate (final concentration) to create the ABTS radical cation (ABTS^•+^). The mixture was then let to stand in the dark at room temperature for 12–16 h prior to use. Following this, a 0.08 mM ABTS^•+^ solution was produced by diluting 4 mL of the reaction mixture with water.

Moreover, 2 mL of 0.08 mM ABTS^•+^ solution was added to 10 mm cuvettes along with 20 µL of the diluted extract or standard, and the mixture was then stirred. Spectrophotometric measurements were taken as soon as the sample was prepared, at 734 nm. Trolox was used to produce a calibration curve in the range of 0.02–1.0 mmol/L for the quantitative analysis of the DPPH^•^ and ABTS^•+^ tests. The data were reported as Trolox equivalent antioxidant capacity (mmol TEAC/g of residue). The FRAP assay was evaluated following Bouzabata et al. [64] by creating a ferric complex of 2,4,6-tris(pyridin-2-yl)-1,3,5-triazine (TPTZ) and Fe^3+^.

To 20 µL of the diluted extract solution or the standard in 10 mm cuvettes, two mL of freshly prepared reagent (0.3123 g TPTZ and 0.5406 g FeCl_3_·6H_2_O in 100 mL of acetate buffer = pH 3.6) were also added. After 60 min, the spectrophotometric measurements were taken at λ = 593 nm. With minor adjustments, the CUPRAC test was carried out in accordance with Bektaşoğlu et al. [66] and Bouzabata et al. [64].

Moreover, 10 mm polystyrene cuvettes were filled with 1 mL of water, 500 µL of copper (II) chloride, 500 µL of neocuproine, 500 µL of ammonium acetate, and 100 µL of methanol (blank), standard, or sample, in that order. After 30 min, spectrophotometric measurements were taken at λ = 450 nm. The external standard method was used to quantitatively analyze the FRAP and CUPRAC assays. Ferrous sulphate in the 0.1–2 mmol range was used, and the results were expressed as mmol Fe^2+^/g of residue.

## 4. Conclusions

The composition of the *C. cardunculus* var. *scolymus* leaf by-products extracted using the four GETs was thoroughly investigated by (HR) LC-ESI-QTOF MS/MS and LC-PDA analyses, which enabled the evaluation of variations in the extraction of different bioactive compounds. Regarding UAE, RSM and BBD indicated that the solvent was the most decisive extraction parameter, especially for luteolin 7-*O*-rutinoside, luteolin 7-*O*-glucoside, apigenin rutinoside, luteolin, chlorogenic acid, and cynaropicrin, the most abundant detected compounds. Noticeably, amplitude and impulse do not have significant influences on the extraction of the target compounds. Among the UAE extracts, the ones with the highest amounts of polyphenols were those extracted with EtOH:H_2_O (50:50, *v*/*v*), while the ones with the lowest amounts were those extracted with 100% water. Interestingly, UAE extracted with 96% EtOH resulted in the highest amount of cynaropicrin. SCO_2_E, a method of choice for non-polar compounds, showed the highest amount of cynaropicrin, a molecule with hydrophobic character and poor water solubility. Consequently, this could suggest that it is a suitable technique for isolating this specific compound. All the DES extracts showed comparable behaviors, while the highest TPC among them was represented by 16DES (choline chloride:levulinic acid). All the SWE extracts demonstrated a trend that was globally higher than those of the other extraction procedures, except flavonoids, which were extracted using 100% water and were not detectable when the temperatures rose. The highest TPC was obtained with EtOH:H_2_O (50:50, *v*/*v*) at the lowest temperature (125 °C), and 96% EtOH also demonstrated better recovery than 100% water. This trend was supported by evaluating the antioxidant activity and radical scavenging activity by FRAP, CUPRAC, DPPH^•^, and ABTS^•+^, which confirmed SWE as an optimal extraction technique for the recovery of phenolic compounds with strong antioxidant activity, such as protocatechuic acid hexoside, pinoresinol hexoside, luteolin, and apigenin. Furthermore, PCA support was crucial not only to confirm the strong activity of SWE but also to note that UAE and DES showed comparable extraction yields for antioxidant compounds. Additionally, it confirmed that EtOH:H_2_O (50:50, *v*/*v*) was the best solvent among UAE and that certain compounds were associated with antioxidant activity, with protocatechuic acid being the compound with the highest one. In conclusion, GETs represent a sustainable approach to extracting intriguing biological compounds with solid antioxidant activity from artichoke leaf by-products. Further studies are necessary to evaluate how these extracts could be applied in the food, pharmaceutical, nutraceutical, and/or cosmetics sectors. They could represent a beneficial approach to carry forward the circular economy, reducing the waste resulting from the agri-food industry.

## Figures and Tables

**Table 1:** *C. cardunculus* var. *scolymus* leaf by-product samples and parameters of the green extraction techniques used.

Sample Code *	Extraction Parameters		
	**Amplitude (%)**	**Impulse (%)**	**Solvent**
1UAE	100	60	100% H_2_O
2UAE	60	20	
3UAE	60	100	
4UAE	20	60	
5UAE	100	100	EtOH:H_2_O (50:50, *v*/*v*)
6UAE	100	20	
7UAE	60	60	
8UAE	60	60	
9UAE	60	60	
10UAE	60	60	
11UAE	60	60	
12UAE	20	100	
13UAE	20	20	
14UAE	100	60	96% EtOH
15UAE	60	20	
16UAE	60	100	
17UAE	20	60	
	**Temperature (°C)**	**Solvent**	
1SWE	125	100% H_2_O	
2SWE	150		
3SWE	175		
4SWE	200		
5SWE	225		
6SWE	250		
7SWE	125	EtOH:H_2_O (50:50, *v*/*v*)	
8SWE	150		
9SWE	175		
10SWE	200		
11SWE	125	96% EtOH	
12SWE	150		
13SWE	175		
14SWE	200		
	**Extraction Solvent**		
1DES	Choline chloride:urea 1:2-H_2_O (80:20, *v*/*v*)		
2DES	Choline chloride:*N*-methyl urea 1:3-H_2_O (80:20, *v*/*v*)		
3DES	Choline chloride:thiourea 1:2-H_2_O (80:20, *v*/*v*)		
4DES	Choline chloride:xylitol 1:1-H_2_O (80:20, *v*/*v*)		
5DES	Choline chloride:sorbitol 1:1-H_2_O (80:20, *v*/*v*)		
6DES	Choline chloride:acetamide 1:2-H_2_O (80:20, *v*/*v*)		
7DES	Choline chloride:butane-1,4-diol 1:2-H_2_O (80:20, *v*/*v*)		
8DES	Choline chloride:ethane-1,2-diol 1:2-H_2_O (80:20, *v*/*v*)		
9DES	Choline chloride:glycerol 1:2-H_2_O (80:20, *v*/*v*)		
10DES	Choline chloride:oxalic acid 1:1-H_2_O (80:20, *v*/*v*)		
11DES	Choline chloride:1,3-dimethylurea 1:2-H_2_O (80:20, *v*/*v*)		
12DES	Choline chloride:maleic acid 1:1-H_2_O (80:20, *v*/*v*)		
13DES	Choline chloride:malic acid 1:1-H_2_O (80:20, *v*/*v*)		
14DES	Choline chloride:malonic acid 1:1-H_2_O (80:20, *v*/*v*)		
15DES	Choline chloride:lactic acid 1:2-H_2_O (80:20, *v*/*v*)		
16DES	Choline chloride:levulinic acid 1:2-H_2_O (80:20, *v*/*v*)		
	**Pressure (bar)**		
SCO_2_	300		

* Extraction technique: UAE, ultrasound-assisted extraction; SWE, subcritical water extraction; DES, deep eutectic solvents; SCO_2_, supercritical CO_2_ extraction.

**Table 2:** Antioxidant activity of *C. cardunculus* var. *scolymus* leaf extracts obtained with selected different green extraction techniques.

Sample Code	TP ^A^	CUPRAC ^B^	FRAP ^B^	DPPH^• C^	ABTS^•+ C^
	(mg GAE/g dp)	(mmol Fe^2+^/g dp)		(mmol TEAC/g dp)	
1UAE	247.62 ± 17.53 ^ac^	4.35 ± 0.49 ^a^	2.18 ± 0.13 ^a^	0.24 ± 0.05 ^a^	0.86 ± 0.06 ^ade^
2UAE	177.30 ± 14.88 ^bf^	5.44 ± 0.29 ^b^	2.02 ± 0.18 ^a^	0.41 ± 0.10 ^beh^	1.23 ± 0.10 ^b^
3UAE	171.62 ± 12.81 ^bf^	4.02 ± 0.62 ^ad^	1.44 ± 0.14 ^b^	0.09 ± 0.02 ^c^	0.80 ± 0.02 ^aef^
4UAE	289.33 ± 60.78 ^a^	7.03 ± 1.10 ^c^	3.07 ± 0.42 ^c^	0.90 ± 0.07 ^d^	1.19 ± 0.05 ^b^
5UAE	215.08 ± 20.81 ^cf^	3.79 ± 0.29 ^a^	2.35 ± 0.32 ^a^	0.52 ± 0.09 ^bf^	1.20 ± 0.10 ^b^
6UAE	124.02 ± 4.83 ^de^	2.92 ± 0.52 ^ed^	1.67 ± 0.06 ^d^	0.38 ± 0.03 ^e^	0.76 ± 0.02 ^c^
7UAE	143.67 ± 23.39 ^bg^	3.15 ± 0.61 ^de^	2.11 ± 0.27 ^a^	0.55 ± 0.03 ^b^	0.87 ± 0.04 ^ad^
8UAE	152.84 ± 12.78 ^bg^	2.63 ± 0.13 ^e^	1.78 ± 0.02 ^e^	0.50 ± 0.09 ^bf^	0.95 ± 0.04 ^d^
9UAE	146.73 ± 39.54 ^befg^	2.13 ± 0.18 ^f^	1.56 ± 0.04 ^b^	0.36 ± 0.05 ^eh^	0.76 ± 0.07 ^ac^
10UAE	192.37 ± 13.35 ^f^	2.53 ± 0.30 ^eg^	1.76 ± 0.08 ^de^	0.35 ± 0.03 ^ah^	0.74 ± 0.07 ^ce^
11UAE	168.56 ± 47.42 ^fg^	2.31 ± 0.07 ^f^	2.43 ± 0.42 ^a^	0.39 ± 0.09 ^afh^	0.79 ± 0.02 ^ef^
12UAE	143.23 ± 16.01 ^dg^	2.18 ± 0.25 ^f^	2.44 ± 0.36 ^a^	0.41 ± 0.06 ^ef^	0.74 ± 0.04 ^cf^
13UAE	170.53 ± 59.47 ^dfg^	2.72 ± 0.51 ^ef^	1.79 ± 0.08 ^de^	0.79 ± 0.10 ^d^	0.95 ± 0.03 ^d^
14UAE	81.00 ± 20.05 ^h^	0.70 ± 0.09 ^g^	0.76 ± 0.12 ^f^	0.28 ± 0.07 ^ae^	0.39 ± 0.05 ^g^
15UAE	61.12 ± 3.23 ^h^	0.32 ± 0.22 ^h^	0.70 ± 0.12 ^f^	0.17 ± 0.04 ^ag^	0.29 ± 0.03 ^h^
16UAE	105.45 ± 62.36 ^g^	0.40 ± 0.27 ^gh^	0.66 ± 0.10 ^f^	0.26 ± 0.06 ^ah^	0.33 ± 0.05 ^eh^
17UAE	27.23 ± 1.62 ^i^	0.05 ± 0.03 ^i^	0.31 ± 0.02 ^g^	0.09 ± 0.02 ^c^	0.17 ± 0.02 ^i^
SCO_2_	109.21 ± 7.57	5.88 ± 0.44	0.93 ± 0.07	0.10 ± 0.02	0.29 ± 0.01
1SWE	399.63 ± 46.67 ^a^	9.24 ± 1.01 ^a^	4.87 ± 0.55 ^a^	1.10 ± 0.01 ^a^	1.04 ± 0.08 ^a^
2SWE	689.46 ± 31.80 ^b^	16.05 ± 1.04 ^b^	7.47 ± 0.63 ^b^	2.31 ± 0.04 ^b^	2.73 ± 0.35 ^b^
3SWE	879.58 ± 21.68 ^c^	34.14 ± 0.67 ^c^	17.11 ± 0.73 ^c^	4.89 ± 0.10 ^c^	6.18 ± 0.24 ^c^
4SWE	1058.76 ± 42.62 ^d^	32.57 ± 0.39 ^d^	20.69 ± 1.07 ^dg^	5.22 ± 0.26 ^cm^	7.15 ± 0.43 ^d^
5SWE	955.01 ± 38.87 ^e^	22.87 ± 1.90 ^e^	16.19 ± 0.72 ^c^	4.92 ± 0.14 ^c^	5.83 ± 0.11 ^c^
6SWE	1346.68 ± 45.33 ^f^	33.05 ± 1.34 ^cd^	20.12 ± 0.15 ^d^	6.37 ± 0.31 ^d^	8.34 ± 0.24 ^e^
7SWE	384.16 ± 29.80 ^a^	10.72 ± 0.68 ^a^	6.28 ± 0.19 ^e^	1.84 ± 0.10 ^e^	1.93 ± 0.19 ^f^
8SWE	639.01 ± 41.39 ^b^	14.29 ± 0.23 ^f^	9.57 ± 0.16 ^f^	3.02 ± 0.15 ^f^	3.48 ± 0.08 ^g^
9SWE	1088.27 ± 45.80 ^dl^	33.40 ±0.84 ^cd^	22.12 ± 1.01 ^g^	5.61 ± 0.07 ^gm^	6.81 ± 0.06 ^h^
10SWE	1433.06 ± 119.52 ^f^	37.47 ± 2.20 ^g^	31.12 ± 0.30 ^h^	8.82 ± 0.56 ^h^	10.19 ± 0.31 ^i^
11SWE	198.32 ± 5.54 ^g^	5.78 ± 0.35 ^h^	3.37 ± 0.21 ^i^	0.84 ± 0.03 ^i^	1.07 ± 0.22 ^a^
12SWE	331.58 ± 11.79 ^h^	8.01 ± 0.21 ^i^	5.98 ± 0.36 ^e^	1.17 ± 0.02 ^l^	1.67 ± 0.09 ^l^
13SWE	591.66 ± 2.97 ^i^	16.34 ± 1.73 ^b^	11.43 ± 0.39 ^l^	2.32 ± 0.04 ^b^	3.33 ± 0.16 ^g^
14SWE	1198.44 ± 93.60 ^l^	30.13 ± 3.07 ^cd^	24.36 ± 0.44 ^m^	5.55 ± 0.19 ^gm^	7.86 ± 0.46 ^de^
1DES	115.57 ± 12.95 ^a^	1.82 ± 0.23 ^a^	1.86 ± 0.11 ^ah^	0.02 ± 0.02 ^a^	1.33 ± 0.11 ^a^
2DES	45.88 ± 5.07 ^b^	1.67 ± 0.03 ^b^	2.16 ± 0.14 ^b^	0.26 ± 0.01 ^b^	0.51 ± 0.08 ^b^
3DES	nm	nm	nm	nm	nm
4DES	24.67 ± 1.98 ^d^	1.57 ± 0.23 ^d^	1.31 ± 0.30 ^d^	0.16 ± 0.01 ^d^	0.55 ± 0.06 ^b^
5DES	50.54 ± 6.37b ^i^	0.37 ± 0.06 ^e^	0.59 ± 0.04 ^e^	0.02 ± 0.00 ^a^	0.19 ± 0.03 ^d^
6DES	31.31 ± 0.49 ^e^	0.99 ± 0.13 ^f^	1.51 ± 0.17 ^d^	0.14 ± 0.02 ^d^	0.57 ± 0.10 ^bf^
7DES	41.45 ± 4.94 ^b^	0.52 ± 0.00 ^g^	0.91 ± 0.04 ^f^	0.19 ± 0.10 ^bd^	0.39 ± 0.01 ^e^
8DES	134.45 ± 3.96 ^f^	2.11 ± 0.03 ^h^	1.53 ± 0.19 ^d^	0.02 ± 0.00 ^a^	0.62 ± 0.03 ^bf^
9DES	21.52 ± 1.48 ^d^	1.19 ± 0.09 ^f^	1.45 ± 0.19 ^d^	0.15 ± 0.01 ^d^	0.39 ± 0.00 ^e^
10DES	93.54 ± 1.48 ^g^	1.14 ± 0.09 ^f^	4.25 ± 0.40 ^g^	2.45 ± 0.05 ^e^	0.42 ± 0.05 ^e^
11DES	63.13 ± 5.88 ^h^	2.27 ± 0.13 ^h^	1.70 ± 0.14 ^ad^	0.28 ± 0.03 ^b^	0.60 ± 0.05 ^bf^
12DES	107.53 ± 4.45 ^a^	1.67 ± 0.10 ^bd^	2.00 ± 0.15 ^bh^	2.83 ± 0.01 ^f^	0.71 ± 0.06 ^l^
13DES	55.10 ± 3.59 ^hi^	2.20 ± 0.06 ^h^	0.33 ± 0.05 ^i^	0.41 ± 0.11 ^g^	0.38 ± 0.02 ^e^
14DES	62.19 ± 6.79 ^h^	0.96 ± 0.14 ^f^	0.18 ± 0.08 ^l^	1.16 ± 0.02 ^h^	0.67 ± 0.05 ^f^
15DES	16.63 ± 1.48 ^l^	2.36 ± 0.31 ^h^	2.55 ± 0.20 ^m^	0.61 ± 0.06 ^i^	0.44 ± 0.09 ^eg^
16DES	123.03 ± 4.66 ^a^	1.96 ± 0.24 ^ah^	2.17 ± 0.09 ^b^	0.38 ± 0.05 ^g^	0.75 ± 0.09 ^f^

All values are expressed per g of dry plant (dp), mean ± SD (*n* = 3); mean values within a column for each green extraction technique with different letters are significantly different at *p* ≤ 0.05. ^A^: total phenolic content (TP) value is expressed as milligrams of gallic acid equivalent (GAE). ^B^: FRAP and CUPRAC values are expressed as the millimolar concentration of Fe^2+^, obtained from a dilution of FeSO_4_ having an equivalent antioxidant capacity to that of the extract. ^C^: DPPH^•^ and ABTS^•+^ values are expressed as the millimolar concentration of TEAC, obtained from a Trolox solution having an antiradical capacity equivalent to that of the extract; nm: not measurable.

## Data Availability

The original contributions presented in the study are included in the article and supplementary material, further inquiries can be directed to the corresponding authors.

## Supplementary Materials

The following supporting information can be downloaded at https://www.mdpi.com/article/10.3390/molecules29204816/s1, Table S1. Compound identification by (HR) LC-ESI-QTOF MS/MS; Table S2abc. Quantification of target compounds by LC-PDA method (mg/g dp); Table S3. Pearson’s correlation coefficients and significance levels; Table S4. Regression coefficients of polynomial function of the most significant response surfaces during UAE; Table S5. Analysis of variance (ANOVA) of the selected modeled responses during UAE. Figure S1. Separation and preparation of *C. cardunculus* var. *scolymus* leaf by-products from edible portions (flower buds and stems). Figure S2. Three-dimensional plots for obtained compounds (luteolin 7-*O*-rutinoside, luteolin 7-*O*-glucoside, apigenin rutinoside, luteolin, and chlorogenic acid) in extracts as a function of UAE process parameters.
