# Supplementary material for: Chemical Profiling and Evaluation of Antioxidant Activity of Artichoke (Cynara cardunculus var. scolymus) Leaf By-Products’ Extracts Obtained with Green Extraction Techniques
Source: Molecules. 2024 Oct 11;29(20):4816. doi: 10.3390/molecules29204816 (PMC11510438; doi:10.3390/molecules29204816)
Supplement: Supplementary file 1 [file molecules-29-04816-s001.zip › molecules-3243996-supplementary.pdf]

Supplementary File

Chemical Profiling and Evaluation of Antioxidant Activity of Artichoke (Cynara cardunculus var. scolymus) Leaf By-Products' Extracts Obtained with Green Extraction Techniques

Valentina Masala, Stela Joki´c , Krunoslav Aladi´c , Maja Molnar , Mattia Casula and Carlo Ignazio Giovanni Tuberoso

Table S1. Compounds identification by (HR) LC-ESI-QTOF MS/MS in *C. cardunculus* var. *scolymus* leaves by-products.

# n°	Rt min	Identity	[M]+/[M+H]+ *m/z*	Molecular formula	MS/MS* *m/z*	Δppm	References	Level
**1**	8.65	Tryptophan	205.0968	$C_{11}H_{12}N_2O_2$	146.0599(90)/118.0643(100)	0.07	[38]	1
**2**	25.87	Cynaropicrin	364.1776 [NH_4^+]	$C_{19}H_{22}O_6$	181.1003(100)	1.44	[39]	1
**# n°**	**Rt min**	**Identity**	**[M-H]- m/z**	**Molecular formula**	**MS/MS* m/z**	**Δppm**	**References**	**Level**
**3**	1.9	Quinic acid	191.0558	$C_7H_{12}O_6$	-	-0.17	[40]	1
**4**	6.64	Protocatechuic acid derivative (hexoside)	315.0718	$C_{13}H_{16}O_9$	153.0175(40)/152.0120(100)	0.46	[41]	3
**5**	8.35	Neochlorogenic acid	353.0879	$C_{16}H_{18}O_9$	191.0556 (100)	0.51	[40, 41, 42]	1
**6**	10.88	Syringic acid hexoside	359.0982	$C_{15}H_{20}O_{10}$	197.0456 (100)/182.0220 (84)	-2.48	[42]	2
**7**	12.84	Chlorogenic acid	353.0878	$C_{16}H_{18}O_9$	191.0561 (100)/ 179.0340 (43)	0.05	[39, 40, 41, 43]	1
**8**	15.42	Coumaroyl-quinic acid	337.0936	$C_{16}H_{18}O_8$	191.0562(89)/ 163.0391(30)	1.15	[42]	2
**9**	16.40	Cynaroscoloside C	471.1876 [FA]	$C_{21}H_{30}O_9$	59.0144 (100)	1.82	[40]	2
**10**	19.04	Cynaroscoloside A/B	473.2035 [FA]	$C_{21}H_{32}O_{12}$	427.1900(100)/ 59.0139 (89)	1.77	[40]	2
**11**	21.24	Luteolin 7-*O*-rutinoside	593.1524	$C_{27}H_{30}O_{15}$	285.0405 (100)/ 284.0340 (30)	1.75	[11, 39, 41, 42, 43]	1
**12**	21.37	Cynaroside (Luteolin 7-*O*-glucoside)	447.0924	$C_{21}H_{20}O_{11}$	285.0396(100)/ 284.0316(32)	-0.91	[39, 40, 41, 42, 43]	1
**13**	22.60	Di-caffeoylquinic acid I	515.1201	$C_{25}H_{24}O_{12}$	191.0563(100) / 353.0779 (35) /135.0452 (62)	0.41	[11, 39, 41, 42, 43]	2
**14**	23.20	Apigenin rutinoside	577.1576	$C_{27}H_{30}O_{14}$	269.0428(100)	2.19	[11, 41, 42]	2
**15**	26.31	Di-caffeoylquinic acid II	561.1622 [FA]	$C_{25}H_{24}O_{12}$	515.1644/191.0554(100)	2.13	[11, 39, 41, 42, 43]	2

**16**	26.73	Pinoresinol hexo-side	519.1864	$C_{26}H_{32}O_{11}$	151.0385(100)/ 357.1323 (27)	0.91	[42, 43]	2
**17**	27.62	Apigenin glucu-ronide	445.0778	$C_{21}H_{18}O_{11}$	269.0448(100)	1.29	[42]	2
**18**	28.10	Luteolin acetyl-glu-coside	489.1044	$C_{23}H_{22}O_{12}$	285.0397(100)/ 284.0307(55)	1.21	[40, 43]	2
**19**	29.16	Luteolin	285.0797	$C_{15}H_{10}O_6$	-	-0.73	[40, 41, 42, 43]	1
**20**	33.27	Apigenin	269.0456	$C_{15}H_{10}O_5$	-	0.07	[42]	1

* [FA]: adduct with formic acid; [NH_4^+]: adduct with ammonia; in parenthesis, the relative intensity; # according to Blaženović [37]. Reference number as listed in the text, section References.

Table S2. Quantification of target compounds by LC-PDA method (mg/g dp).

(a)

Compounds §		1UAE	2UAE	3UAE	4UAE	5UAE	6UAE	7UAE	8UAE	9UAE
**Flavonoids**										
Luteolin 7-*O*-rutinoside	11	0.16 ± 0.02a	0.15 ± 0.01a	0.15 ± 0.01a	0.16 ± 0.01a	0.97 ± 0.09b	0.60 ± 0.04c	0.57 ± 0.07c	0.68 ± 0.07c	0.64 ± 0.06cf
Luteolin 7-*O*-glucoside	12	0.16 ± 0.01a	0.16 ± 0.02a	0.16 ± 0.01a	0.16 ± 0.01a	1.53 ± 0.12b	1.01 ± 0.08c	1.10 ± 0.08cd	1.20 ± 0.06d	1.15 ± 0.09cd
Apigenin rutinoside [a]	14	0.15 ± 0.02a	nd	nd	0.16 ± 0.02a	0.35 ± 0.04b	0.26 ± 0.03c	0.26 ± 0.02c	0.28 ± 0.02c	0.27 ± 0.03c
Apigenin glucuronide [a]	17	nd	nd	nd	nd	0.23 ± 0.02a	0.20 ± 0.01a	0.21 ± 0.02a	0.21 ± 0.02a	0.21 ± 0.01a
Luteolin acetyl-glucoside [b]	18	nd	nd	nd	nd	nd	nd	nd	nd	nd
Luteolin	19	0.19 ± 0.01a	0.18 ± 0.02a	0.19 ± 0.02a	0.21 ± 0.01ab	0.25 ± 0.03b	0.33 ± 0.03c	0.47 ± 0.05d	0.35 ± 0.04ce	0.36 ± 0.03ce
Apigenin	20	0.17 ± 0.02ab	0.17 ± 0.02ab	nd	0.17 ± 0.01ab	nd	0.19 ± 0.01a	0.21 ± 0.02a	nd	0.19 ± 0.02a
Other flavonoids [b]		nd	nd	nd	nd	1.64 ± 0.15a	1.19 ± 0.12b	1.29 ± 0.13bc	1.39 ± 0.13c	1.34 ± 0.12c
**Total**		0.83 ± 0.07a	0.67 ± 0.07b	0.50 ±0.05c	0.87 ± 0.07a	4.98 ± 0.40df	3.79 ± 0.30eg	4.11 ± 0.39eg	4.10 ± 0.41eg	4.16 ± 0.33eg
**Hydroxycynnamic acids**										
Chlorogenic acid	7	nd	nd	nd	0.02 ± 0.00a	0.63 ± 0.05b	0.34 ± 0.03ce	0.37 ± 0.04cd	0.42 ± 0.03d	0.35 ± 0.02c
Coumaroyl-quinic acid [c]	8	nd	nd	nd	nd	0.10 ± 0.02a	0.08 ± 0.01a	0.07 ± 0.01a	0.09 ± 0.01a	0.09 ± 0.01a
Di-caffeoylquinic acid I [c]	13	0.05 ± 0.01a	0.02 ± 0.00b	0.03 ± 0.00c	0.07 ± 0.01a	0.02 ± 0.00b	0.01 ± 0.00d	0.01 ± 0.00d	0.02 ± 0.01b	0.01 ± 0.00d
Di-caffeoylquinic acid II [c]	15	0.01 ± 0.00a	nd	0.01 ± 0.00a	0.02 ± 0.00b	0.03 ± 0.01b	0.02 ± 0.00b	0.02 ± 0.00b	0.02 ± 0.00b	0.02 ± 0.00b
Other hydroxycinnamic acids [c]		nd	nd	nd	0.01 ± 0.00a	0.41 ± 0.03b	0.27 ± 0.03c	0.25 ± 0.02c	0.26 ± 0.03c	0.30 ± 0.03cd
**Total**		0.06 ± 0.01a	0.02 ± 0.00b	0.04 ± 0.00c	0.12 ± 0.01d	1.18 ± 0.06e	0.72 ± 0.05f	0.71 ± 0.03f	0.80 ± 0.07f	0.77 ± 0.08f
**Hydroxybenzoic acids**										
Protocatechuic acid hexoside [d]	4	nd	0.05 ± 0.01a	0.02 ± 0.00b	0.04 ± 0.00c	nd	nd	nd	nd	nd
Other hydroxybenzoic acids [d]		0.09 ± 0.01a	0.09 ± 0.01a	0.06 ± 0.01b	0.17 ± 0.02c	1.06 ± 0.09d	0.96 ± 0.10d	0.95 ± 0.08d	1.01 ± 0.11d	1.01 ± 0.09d
**Total**		0.09 ± 0.01a	0.15 ± 0.02b	0.08 ± 0.01a	0.21 ± 0.01c	1.06 ± 0.09d	0.96 ± 0.10d	0.95 ± 0.08d	1.01 ± 0.11d	1.01 ± 0.09d
**Others**										
Tryptophan	1	tr	tr	nd	tr	0.02 ± 0.00a	0.02 ± 0.01a	0.02 ± 0.00a	0.02 ± 0.00a	0.02 ± 0.00a
Cynaroscoloside A/B [e]	10	tr	tr	nd	tr	tr	tr	tr	tr	tr
Cynaropicrin	2	nd	tr	nd	nd	2.72 ± 0.22af	1.70 ± 0.14bc	1.75 ± 0.16bc	2.01 ± 0.18be	1.46 ± 0.11cd
Pinoresinol hexoside [f]	16	0.16 ± 0.02a	0.08 ± 0.01b	0.11 ± 0.01c	0.22 ± 0.02d	0.07 ± 0.02b	0.06 ± 0.01b	0.07 ± 0.01b	0.07 ± 0.01b	0.07 ± 0.00b
Others [e]		0.02 ± 0.00a	nd	nd	0.02 ± 0.01af	0.42 ± 0.03b	0.36 ± 0.02c	0.57 ± 0.05d	0.36 ± 0.03c	0.45 ± 0.05b
**Total**		0.18 ± 0.02a	0.09 ± 0.01b	0.11 ± 0.01b	0.25 ± 0.03c	3.23 ± 0.30d	2.14 ± 0.20ef	2.42 ± 0.19eh	2.46 ± 0.25eh	2.00 ± 0.16fg
**TOTAL PHENOLS**		0.98 ± 0.05a	0.84 ± 0.06b	0.62 ± 0.03c	1.20 ± 0.06di	7.22 ± 0.58eh	5.47 ± 0.49fg	5.77 ± 0.46fg	5.92 ± 0.36fg	5.94 ± 0.53fg

Compounds §		10UAE	11UAE	12UAE	13UAE	14UAE	15UAE	16UAE	17UAE	SCO_2
**Flavonoids**										
Luteolin 7-*O*-rutinoside	11	0.58 ± 0.04c	0.66 ± 0.07cf	0.64 ± 0.05cf	0.68 ± 0.05cf	0.25 ± 0.03d	0.21 ± 0.02e	0.25 ± 0.01d	0.20 ± 0.01e	0.74 ± 0.06f
Luteolin 7-*O*-glucoside	12	1.01 ± 0.08c	1.15 ± 0.11cd	1.16 ± 0.06d	1.21 ± 0.10de	0.32 ± 0.02e	0.25 ± 0.02f	0.31 ± 0.02e	0.24 ± 0.01f	0.78 ± 0.04g
Apigenin rutinoside [a]	14	0.26 ± 0.01c	0.27 ± 0.02c	0.27 ± 0.01c	0.28 ± 0.02c	0.17 ± 0.02a	0.16 ± 0.01a	nd	0.17 ± 0.01a	nd
Apigenin glucuronide [a]	17	0.20 ± 0.02a	0.20 ± 0.02a	0.21 ± 0.01a	0.21 ± 0.01a	nd	nd	nd	nd	nd
Luteolin acetyl-glucoside [b]	18	nd	nd	nd	nd	nd	nd	nd	nd	nd
Luteolin	19	0.34 ± 0.03c	0.33 ± 0.02c	0.41 ± 0.03de	0.37 ± 0.02c	0.16 ± 0.02a	0.16 ± 0.02a	nd	nd	nd
Apigenin	20	0.19 ± 0.01a	0.19 ± 0.02a	0.20 ± 0.02a	0.20 ± 0.01a	nd	0.15 ± 0.01b	nd	nd	nd
Other flavonoids [b]		1.21 ± 0.11bc	1.34 ± 0.10bc	1.35 ± 0.12bc	1.35 ± 0.13bc	0.19 ± 0.01d	0.17 ± 0.02d	0.18 ± 0.02d	0.17 ± 0.02d	nd
**Total**		3.78 ± 0.19e	4.13 ± 0.21eg	4.25 ± 0.34efg	4.30 ± 0.22g	1.08 ± 0.11h	1.11 ± 0.06h	0.75 ± 0.04a	0.79 ± 0.08a	1.53 ± 0.14i
**Hydroxycynnamic acids**										
Chlorogenic acid	7	0.29 ± 0.02e	0.35 ± 0.04cde	0.33 ± 0.02ce	0.42 ± 0.03d	0.09 ± 0.01f	0.06 ± 0.01g	0.10 ± 0.01f	0.05 ± 0.00g	nd
Coumaroyl-quinic acid [c]	8	0.08 ± 0.00a	0.09 ± 0.01a	0.08 ± 0.01a	0.09 ± 0.01a	0.01 ± 0.00b	nd	0.01 ± 0.00b	nd	nd
Di-caffeoylquinic acid I [c]	13	0.01 ± 0.00d	0.01 ± 0.00d	0.01 ± 0.00d	0.02 ± 0.01bd	nd	nd	nd	nd	nd
Di-caffeoylquinic acid II [c]	15	0.02 ± 0.01b	0.02 ± 0.00b	0.02 ± 0.00b	0.02 ± 0.00b	nd	nd	nd	nd	nd
Other hydroxycinnamic acids [c]		0.28 ± 0.03c	0.27 ± 0.01c	0.25 ± 0.02c	0.34 ± 0.02d	0.05 ± 0.01ef	0.04 ± 0.00e	0.06 ± 0.01f	0.03 ± 0.00g	0.08 ± 0.00h
**Total**		0.69 ± 0.05f	0.74 ± 0.06f	0.70 ± 0.07f	0.90 ± 0.08g	0.16 ± 0.02h	0.09 ± 0.02ad	0.16 ± 0.01h	0.09 ± 0.02ad	0.08 ± 0.01a
**Hydroxybenzoic acids**										
Protocatechuic acid hexoside [d]	4	nd	nd	nd	nd	nd	nd	nd	nd	nd
Other hydroxybenzoic acids [d]		0.93 ± 0.05d	1.03 ± 0.09d	1.01 ± 0.08d	1.03 ± 0.05d	0.24 ± 0.02e	0.17 ± 0.02c	0.26 ± 0.01e	0.15 ± 0.02c	nd
**Total**		0.93 ± 0.05d	1.03 ± 0.09d	1.01 ± 0.08d	1.03 ± 0.06d	0.24 ± 0.02ce	0.17 ± 0.02b	0.26 ± 0.01e	0.15 ± 0.02b	nd
**Others**										
Tryptophan	1	0.02 ± 0.00a	0.02 ± 0.01a	0.02 ± 0.00a	0.02 ± 0.00a	tr	tr	tr	tr	nd
Cynaroscoloside A/B [e]	10	tr	tr	tr	tr	tr	0.03 ± 0.01a	0.09 ± 0.04b	0.02 ± 0.00a	8.22 ± 0.74c
Cynaropicrin	2	1.24 ± 0.12d	1.35 ± 0.11d	1.68 ± 0.14c	2.34 ± 0.20ae	2.32 ± 0.19ae	2.64 ± 0.22af	3.02 ± 0.29f	2.61 ± 0.21af	48.33 ± 2.42g
Pinoresinol hexoside [f]	16	0.06 ± 0.01b	0.07 ± 0.01b	0.07 ± 0.01b	0.07 ± 0.01b	0.02 ± 0.00e	0.01 ± 0.00f	0.02 ± 0.00e	0.01 ± 0.00f	nd
Others [e]		0.44 ± 0.03b	0.38 ± 0.04bc	0.57 ± 0.06d	0.39 ± 0.02bc	0.05 ± 0.01e	0.04 ± 0.01ef	0.06 ± 0.01e	0.03 ± 0.00f	nd
**Total**		1.76 ± 0.14g	1.81 ± 0.15fg	2.34 ± 0.19e	2.82 ± 0.30efh	2.39 ± 0.24he	2.71 ± 0.25edh	3.19 ± 0.28df	2.67 ± 0.13h	56.54 ± 2.83i
**TOTAL PHENOLS**		5.40 ± 0.27f	5.90 ± 0.30fg	5.96 ± 0.42fg	6.23 ± 0.50gh	1.48 ± 0.07il	1.37 ± 0.11il	1.17 ± 0.10il	1.03 ± 0.09a	1.61 ± 0.14l

§ peak number as reported in Table S1. [a] expressed as apigenin 7-*O*-glucoside equivalents; [b] luteolin 7-*O*-glucoside equivalents; [c] expressed as chlorogenic acid equivalents; [d] expressed as protocatechuic acid equivalents; [e] expressed as cynaropicrin equivalents; [f] expressed as pinoresinol equivalents. nd: not detected. tr: traces. Data are given as mean ± standard deviation ($n = 3$). Mean values within a line with different letters are significantly different (homogenous groups) at $p \leq 0.05$.

(b)

Compounds §		1SWE	2SWE	3SWE	4SWE	5SWE	6SWE	7SWE	8SWE	9SWE	10SWE	11SWE	12SWE	13SWE	14SWE
**Flavonoids**															
Luteolin 7-*O*-rutinoside	11	1.45 ± 0.12a	1.04 ± 0.05b	1.22 ± 0.08bc	nd	nd	nd	3.49 ± 0.33d	3.01 ± 0.21d	2.50 ± 0.20e	1.04 ± 0.08b	2.00 ± 0.14f	2.43 ± 0.20e	2.62 ± 0.18e	2.14 ± 0.19ef
Luteolin 7-*O*-glucoside	12	1.31 ± 0.11a	1.99 ± 0.16b	1.33 ± 0.07a	nd	nd	nd	7.50 ± 0.38c	6.76 ± 0.34d	4.60 ± 0.37ef	1.18 ± 0.10a	4.15 ± 0.37eh	5.12 ± 0.46f	5.33 ± 0.32g	3.57 ± 0.29h
Apigenin rutinoside [a]	14	0.53 ± 0.04af	0.49 ± 0.05ab	0.43 ± 0.03b	nd	nd	nd	0.91 ± 0.08c	0.81 ± 0.06d	0.71 ± 0.05e	0.46 ± 0.03ab	0.59 ± 0.04f	0.61 ± 0.05f	0.61 ± 0.04f	0.59 ± 0.03f
Apigenin glucuronide [a]	17	nd	0.40 ± 0.02a	nd	nd	nd	nd	1.00 ± 0.09b	0.96 ± 0.08b	0.73 ± 0.06c	0.37 ± 0.02d	0.82 ± 0.04c	1.30 ± 0.10e	1.38 ± 0.09e	1.04 ± 0.06b
Luteolin acetyl-glucoside [b]	18	nd	nd	nd	nd	nd	nd	0.48 ± 0.02a	nd	nd	nd	nd	0.56 ± 0.03b	0.40 ± 0.04c	nd
Luteolin	19	1.24 ± 0.07a	2.08 ± 0.17b	0.56 ± 0.04c	nd	nd	nd	0.56 ± 0.05c	0.92 ± 0.07d	1.88 ± 0.15bg	4.20 ± 0.33e	0.50 ± 0.04c	0.70 ± 0.04f	1.68 ± 0.10g	3.55 ± 0.25h
Apigenin	20	nd	0.43 ± 0.03ab	nd	nd	nd	nd	nd	0.41 ± 0.04ab	0.40 ± 0.03a	0.47 ± 0.02b	nd	0.47 ± 0.03b	0.49 ± 0.05b	0.48 ± 0.04b
Other flavonoids [b]		1.18 ± 0.07a	1.15 ± 0.10a	0.36 ± 0.03b	nd	nd	nd	2.40 ± 0.14c	1.99 ± 0.18d	0.72 ± 0.07e	0.37 ± 0.02b	1.53 ± 0.08f	1.85 ± 0.15d	0.87 ± 0.07g	0.42 ± 0.03b
**Total**		5.72 ± 0.51a	7.59 ± 0.61b	3.90 ± 0.27c	nd	nd	nd	16.36 ± 1.47d	14.87 ± 1.18df	11.55 ± 0.69ef	8.09 ± 0.73be	9.59 ± 0.96e	13.04 ± 1.04f	13.38 ± 0.80f	11.79 ± 1.06ef
**Hydroxycynnamic acids**															
Chlorogenic acid	7	0.08 ± 0.01a	1.20 ± 0.10b	1.06 ± 0.09b	0.64 ± 0.05c	0.30 ± 0.02d	nd	3.28 ± 0.20e	2.46 ± 0.25f	1.67 ± 0.16gh	1.05 ± 0.05b	1.56 ± 0.11g	2.11 ± 0.15fh	1.90 ± 0.10h	0.45 ± 0.02i
Coumaroyl-quinic acid [c]	8	0.05 ± 0.00a	0.12 ± 0.01b	0.14 ± 0.01b	0.08 ± 0.00c	nd	nd	0.20 ± 0.02d	0.19 ± 0.02d	0.19 ± 0.01d	0.13 ± 0.01b	0.17 ± 0.02d	0.30 ± 0.03e	0.27 ± 0.03e	0.07 ± 0.01c
Di-caffeoylquinic acid I [c]	13	nd	0.08 ± 0.01a	0.03 ± 0.00b	0.03 ± 0.00b	0.03 ± 0.00b	0.08 ± 0.01a	0.04 ± 0.01bd	0.04 ± 0.01bd	0.03 ± 0.01bcd	0.02 ± 0.00c	0.05 ± 0.01d	0.04 ± 0.01bd	0.03 ± 0.00b	nd
Di-caffeoylquinic acid II [c]	15	nd	nd	nd	nd	0.04 ± 0.00a	nd	nd	nd	0.05 ± 0.01a	nd	0.04 ± 0.01a	0.05 ± 0.01a	0.04 ± 0.01a	nd
Other hydroxycinnamic acids [c]		0.26 ± 0.03a	0.13 ± 0.01b	0.32 ± 0.02c	0.53 ± 0.04d	0.30 ± 0.03ac	nd	0.75 ± 0.04e	0.51 ± 0.03d	0.47 ± 0.02dg	0.64 ± 0.05f	0.44 ± 0.03g	0.46 ± 0.05dg	0.46 ± 0.04dg	0.73 ± 0.04ef
**Total**		0.40 ± 0.02a	1.52 ± 0.08b	1.56 ± 0.11b	1.28 ± 0.10c	0.67 ± 0.05d	0.08 ± 0.01e	4.26 ± 0.30f	3.19 ± 0.22g	2.42 ± 0.17h	1.84 ± 0.15i	2.25 ± 0.18h	2.96 ± 0.27gl	2.70 ± 0.24hl	1.26 ± 0.10c
**Hydroxybenzoic acids**															
Protocatechuic acid hexoside [d]	4	0.29 ± 0.03a	0.78 ± 0.06b	0.92 ± 0.09c	0.59 ± 0.05d	nd	nd	0.16 ± 0.02e	0.67 ± 0.07bd	0.78 ± 0.06b	0.18 ± 0.01e	0.09 ± 0.00f	0.22 ± 0.01g	0.70 ± 0.05b	0.28 ± 0.03a
Other hydroxybenzoic acids [d]		0.90 ± 0.06a	1.78 ± 0.13b	1.16 ± 0.10c	1.70 ± 0.15b	1.46 ± 0.13d	1.82 ± 0.18bf	2.61 ± 0.23e	1.31 ± 0.12cd	1.57 ± 0.09b	2.04 ± 0.10f	2.04 ± 0.16f	1.26 ± 0.11c	1.39 ± 0.08d	1.46 ± 0.12d
**Total**		1.20 ± 0.10a	2.56 ± 0.15bf	2.08 ± 0.12ce	2.29 ± 0.18bc	1.46 ± 0.15d	1.82 ± 0.18e	2.77 ± 0.25f	1.98 ± 0.18ce	2.36 ± 0.21bcf	2.21 ± 0.18bc	2.12 ± 0.17fc	1.49 ± 0.13d	2.08 ± 0.17ce	1.74 ± 0.14e
**Others**															
Tryptophan	1	nd	0.01 ± 0.00a	nd	nd	nd	nd	0.02 ± 0.00b	nd	nd	nd	nd	nd	nd	nd
Cynaroscoloside A/B [e]	10	tr	nd	tr	tr	nd	nd	0.17 ± 0.02a	tr	tr	tr	tr	tr	tr	tr
Cynaropicrin	2	1.33 ± 0.12a	0.82 ± 0.07b	tr	tr	nd	nd	5.41 ± 0.43c	2.20 ± 0.18d	0.53 ± 0.04e	nd	3.72 ± 0.26f	3.66 ± 0.22f	3.21 ± 0.32f	0.65 ± 0.04g
Pinoresinol hexoside [f]	16	0.07 ± 0.01a	0.28 ± 0.01b	0.11 ± 0.01c	0.16 ± 0.02d	0.15 ± 0.01d	0.09 ± 0.00e	0.17 ± 0.02d	0.16 ± 0.02d	0.14 ± 0.01d	0.24 ± 0.02f	0.14 ± 0.01d	0.14 ± 0.01d	0.16 ± 0.02d	0.22 ± 0.02f
Others [e]		0.61 ± 0.03af	0.62 ± 0.06af	1.02 ± 0.09b	2.23 ± 0.11c	5.92 ± 0.47d	10.10 ± 0.90e	0.67 ± 0.6a	0.54 ± 0.05f	0.67 ± 0.07a	1.59 ± 0.14g	0.23 ± 0.02hi	0.22 ± 0.01h	0.21 ± 0.02h	0.28 ± 0.03i
**Total**		2.00 ± 0.18a	1.72 ± 0.14a	1.13 ± 0.11b	2.40 ± 0.20c	6.07 ± 0.61d	10.20 ± 0.71e	6.44 ± 0.45d	2.91 ± 0.17f	1.34 ± 0.07g	1.84 ± 0.15a	4.09 ± 0.33h	4.02 ± 0.36h	3.58 ± 0.32h	1.15 ± 0.09b
**TOTAL PHENOLS**		7.31 ± 0.73a	11.68 ± 1.05b	7.54 ± 0.68a	3.58 ± 0.25c	2.12 ± 0.13d	1.90 ± 0.11d	23.39 ± 1.87e	20.05 ± 1.80f	16.32 ± 1.47gi	12.14 ± 0.61b	13.97 ± 1.12h	17.48 ± 1.25g	18.16 ± 1.63gf	14.79 ± 1.48i

§ peak number as reported in Table S1. [a] expressed as apigenin 7-*O*-glucoside equivalents; [b] luteolin 7-*O*-glucoside equivalents; [c] expressed as chlorogenic acid equivalents; [d] expressed as protocatechuic acid equivalents; [e] expressed as cynaropicrin equivalents; [f] expressed as pinoresinol equivalents. nd: not detected. tr: traces. Data are given as mean ± standard deviation ($n = 3$). Mean values within a line with different letters are significantly different (homogenous groups) at $p \leq 0.05$.

(c)

Compounds §		1DES	2DES	3DES	4DES	5DES	6DES	7DES	8DES
**Flavonoids**									
Luteolin 7-*O*-rutinoside	11	0.55 ± 0.04a	1.16 ± 0.10bd	1.22 ± 0.12b	0.82 ± 0.07c	0.80 ± 0.08c	0.95 ± 0.09cd	0.89 ± 0.07c	0.97 ± 0.08cd
Luteolin 7-*O*-glucoside	12	0.71 ± 0.04a	1.66 ± 0.08b	1.79 ± 0.13b	1.12 ± 0.11c	1.13 ± 0.09c	1.34 ± 0.13cd	1.27 ± 0.06ce	1.42 ± 0.07dg
Apigenin rutinoside [a]	14	0.41 ± 0.03ae	0.45 ± 0.05acde	0.59 ± 0.06be	0.38 ± 0.03a	0.41 ± 0.02a	0.51 ± 0.04bcde	0.48 ± 0.02dh	0.41 ± 0.04ae
Apigenin glucuronide [a]	17	0.36 ± 0.04a	0.39 ± 0.03ab	0.43 ± 0.02b	0.36 ± 0.02a	0.36 ± 0.04a	0.38 ± 0.03ab	0.37 ± 0.04ab	0.37 ± 0.04ab
Luteolin acetyl-glucoside [b]	18	0.34 ± 0.03a	0.34 ± 0.02a	0.35 ± 0.04a	0.34 ± 0.02a	0.34 ± 0.03a	0.34 ± 0.03a	0.34 ± 0.03a	0.34 ± 0.02a
Luteolin	19	nd	0.37 ± 0.03a	0.41 ± 0.04a	0.35 ± 0.02a	0.36 ± 0.04a	0.38 ± 0.03a	0.39 ± 0.02a	0.38 ± 0.02a
Apigenin	20	nd	nd	nd	nd	nd	nd	nd	nd
Other flavonoids [b]		1.29 ± 0.06a	2.07 ± 0.17b	2.14 ± 0.19b	1.56 ± 0.16c	1.63 ± 0.08c	1.79 ± 0.14cd	1.62 ± 0.15c	1.72 ± 0.12cd
**Total**		3.65 ± 0.33a	6.45 ± 0.58bdf	6.92 ± 0.42bf	4.92 ± 0.39c	5.02 ± 0.40c	5.69 ± 0.57cd	5.37 ± 0.48ce	5.61 ± 0.45ce
**Hydroxycynnamic acids**									
Chlorogenic acid	7	0.04 ± 0.00a	0.62 ± 0.06bf	0.62 ± 0.03bf	0.39 ± 0.04ce	0.47 ± 0.05c	0.45 ± 0.03c	0.31 ± 0.02d	0.39 ± 0.02e
Coumaroyl-quinic acid [c]	8	nd	0.04 ± 0.00a	0.07 ± 0.01be	0.04 ± 0.00a	0.04 ± 0.00a	0.04 ± 0.00a	0.04 ± 0.00a	0.05 ± 0.01ab
Di-caffeoylquinic acid I [c]	13	nd	nd	nd	nd	nd	nd	nd	nd
Di-caffeoylquinic acid II [c]	15	nd	nd	nd	nd	nd	nd	nd	nd
Other hydroxycinnamic acids [c]		0.04 ± 0.00a	0.12 ± 0.01b	0.12 ± 0.01b	0.19 ± 0.01c	0.22 ± 0.02cd	0.26 ± 0.03de	0.23 ± 0.02d	0.24 ± 0.02de
**Total**		0.07 ± 0.01a	0.78 ± 0.08b	0.81 ± 0.04b	0.62 ± 0.05cd	0.73 ± 0.07bc	0.75 ± 0.06be	0.58 ± 0.05d	0.68 ± 0.03ce
**Hydroxybenzoic acids**									
Protocatechuic acid hexoside [d]	4	nd	0.05 ± 0.01a	nd	nd	nd	nd	nd	nd
Other hydroxybenzoic acids [d]		0.66 ± 0.07ac	0.67 ± 0.05ad	0.22 ± 0.02b	0.55 ± 0.05c	0.54 ± 0.04c	0.78 ± 0.07de	0.80 ± 0.04e	0.78 ± 0.05e
**Total**		0.66 ± 0.07ac	0.72 ± 0.06ad	0.22 ± 0.02b	0.55 ± 0.05c	0.54 ± 0.05cg	0.78 ± 0.07ad	0.80 ± 0.04d	0.78 ± 0.05ad
**Others**									
Tryptophan	1	nd	nd	0.01 ± 0.00a	tr	tr	0.01 ± 0.00a	0.01 ± 0.00a	0.01 ± 0.00a
Cynaroscoloside A/B [e]	10	tr	tr	tr	tr	tr	tr	tr	tr
Cynaropicrin	2	tr	1.31 ± 0.09a	nd	0.84 ± 0.06b	0.73 ± 0.04c	2.35 ± 0.21dfg	3.14 ± 0.25e	2.18 ± 0.17dg
Pinoresinol hexoside [f]	16	0.02 ± 0.00a	0.04 ± 0.00b	0.07 ± 0.01c	0.02 ± 0.00a	0.02 ± 0.00a	0.04 ± 0.00b	0.05 ± 0.01bc	0.04 ± 0.00b
Others [e]		0.14 ± 0.01a	0.13 ± 0.01a	0.24 ± 0.02b	0.16 ± 0.02ad	0.16 ± 0.02ad	0.18 ± 0.02cd	0.32 ± 0.03e	0.25 ± 0.01b
**Total**		0.17 ± 0.02a	1.48 ± 0.12b	0.32 ± 0.03c	1.02 ± 0.08d	0.92 ± 0.07d	2.58 ± 0.18e	3.52 ± 0.28f	2.47 ± 0.17e
**TOTAL PHENOLS**		4.39 ± 0.35a	7.95 ± 0.72b	7.95 ± 0.64b	6.09 ± 0.55c	6.29 ± 0.57d	7.23 ± 0.65b	6.75 ± 0.61bc	7.07 ± 0.57bc

Compounds §		9DES	10DES	11DES	12DES	13DES	14DES	15DES	16DES
**Flavonoids**									
Luteolin 7-*O*-rutinoside	11	0.95 ± 0.10cd	1.03 ± 0.05d	1.10 ± 0.11bd	1.28 ± 0.06b	1.10 ± 0.09bd	0.86 ±0.06c	1.14 ± 0.08bd	1.24 ± 0.11b
Luteolin 7-*O*-glucoside	12	1.39 ± 0.07de	2.11 ± 0.19fi	1.55 ± 0.16bdg	2.30 ± 0.21f	1.59 ± 0.11gh	1.24 ± 0.09c	1.70 ± 0.12bh	1.84 ± 0.15bi
Apigenin rutinoside [a]	14	0.41 ± 0.03ae	0.56 ± 0.06be	0.45 ± 0.02deh	0.51 ± 0.03bde	0.49 ± 0.05eh	0.42 ± 0.04ah	0.58 ± 0.04b	0.54 ± 0.05bd
Apigenin glucuronide [a]	17	0.37 ± 0.02a	0.34 ± 0.02a	0.38 ± 0.03ab	0.37 ± 0.04ab	0.39 ± 0.04ab	0.36 ± 0.02a	0.40 ± 0.03b	0.40 ± 0.04b
Luteolin acetyl-glucoside [b]	18	0.34 ± 0.02a	nd	0.34 ± 0.03a	0.35 ± 0.04a	0.34 ± 0.03a	0.34 ± 0.03a	0.35 ± 0.02a	0.35 ± 0.04a
Luteolin	19	0.36 ± 0.03a	0.41 ± 0.02a	0.39 ± 0.03a	0.40 ± 0.04a	0.39 ± 0.02a	0.37 ± 0.03a	0.40 ± 0.04a	0.40 ± 0.03a
Apigenin	20	nd	nd	nd	nd	nd	nd	nd	nd
Other flavonoids [b]		1.68 ± 0.15cd	1.58 ± 0.16c	2.01 ± 0.20b	2.12 ± 0.11b	1.98 ± 0.16bd	1.74 ± 0.10c	2.14 ± 0.15b	2.28 ± 0.14b
**Total**		5.50 ± 0.39ce	6.04 ± 0.54bdeg	6.22 ± 0.44bdeg	7.33 ± 0.59f	6.28 ± 0.56bg	5.32 ± 0.48cg	6.72 ± 0.54bf	7.05 ± 0.49bf
**Hydroxycynnamic acids**									
Chlorogenic acid	7	0.39 ± 0.04ce	0.64 ± 0.04b	0.60 ± 0.03bf	0.58 ± 0.06bf	0.58 ± 0.05bf	0.38 ± 0.04ce	0.68 ± 0.07b	0.54 ± 0.05f
Coumaroyl-quinic acid [c]	8	0.04 ± 0.00a	0.11 ± 0.01c	0.04 ± 0.00a	0.15 ± 0.02d	0.08 ± 0.01e	0.08 ± 0.00e	0.09 ± 0.01e	0.09 ± 0.01e
Di-caffeoylquinic acid I [c]	13	nd	0.03 ± 0.00a	nd	0.02 ± 0.00b	0.02 ± 0.00b	nd	0.02 ± 0.00b	0.02 ± 0.00b
Di-caffeoylquinic acid II [c]	15	nd	nd	nd	nd	nd	0.04a	nd	nd
Other hydroxycinnamic acids [c]		0.24 ± 0.02de	0.11 ± 0.01b	0.24 ± 0.01d	0.22 ± 0.02cd	0.25 ± 0.03de	0.08 ± 0.01a	0.28 ± 0.02ef	0.33 ± 0.03f
**Total**		0.67 ± 0.06de	0.88 ± 0.09b	0.88 ± 0.08b	0.97 ± 0.05f	0.94 ± 0.09f	0.58 ± 0.06d	1.07 ± 0.08f	0.97 ± 0.07f
**Hydroxybenzoic acids**									
Protocatechuic acid hexoside [d]	4	nd	nd	nd	nd	nd	nd	nd	nd
Other hydroxybenzoic acids [d]		0.74 ± 0.07ae	1.11 ± 0.07f	0.73 ± 0.07ae	0.32 ± 0.03g	0.47 ± 0.02h	0.34 ± 0.02g	0.23 ± 0.01b	1.67 ± 0.13i
**Total**		0.74 ± 0.07ad	1.11 ± 0.07e	0.73 ± 0.07ad	0.32 ± 0.03f	0.47 ± 0.02g	0.34 ± 0.02f	0.23 ± 0.01b	1.67 ± 0.13h
**Others**									
Tryptophan	1	0.01 ± 0.00a	tr	tr	0.01 ± 0.00a	0.01 ± 0.00a	tr	0.01 ± 0.00a	tr
Cynaroscoloside A/B [e]	10	tr	tr	tr	tr	tr	tr	tr	tr
Cynaropicrin	2	1.40 ± 0.13a	2.76 ± 0.22fg	2.28 ± 0.16dg	2.10 ± 0.13d	2.51 ± 0.23gi	0.42 ± 0.02h	2.70 ± 0.24fil	3.19 ± 0.26el
Pinoresinol hexoside [f]	16	0.04 ± 0.00b	0.04 ± 0.00b	0.04 ± 0.00b	0.06 ± 0.01c	0.04 ± 0.00b	0.03 ± 0.00d	0.05 ± 0.01bc	0.05 ± 0.01bc
Others [e]		0.27 ± 0.03b	0.48 ± 0.02f	0.02 ± 0.00g	0.07 ± 0.01h	0.10 ± 0.01i	0.18 ± 0.02d	0.29 ± 0.02eb	14.73 ± 1.18l
**Total**		1.72 ± 0.15b	3.29 ± 0.30f	2.34 ± 0.19e	2.24 ± 0.20e	2.65 ± 0.27eh	0.64 ± 0.05g	3.05 ± 0.27fh	17.97 ± 1.62i
**TOTAL PHENOLS**		6.92 ± 0.48bc	8.03 ± 0.64b	7.83 ± 0.70b	8.62 ± 0.78be	7.69 ± 0.62b	6.24 ± 0.56c	8.01 ± 0.64b	9.69 ± 0.87e

§ peak number as reported in Table S1. [a] expressed as apigenin 7-*O*-glucoside equivalents; [b] luteolin 7-*O*-glucoside equivalents; [c] expressed as chlorogenic acid equivalents; [d] expressed as protocatechuic acid equivalents; [e] expressed as cynaropicrin equivalents; [f] expressed as pinoresinol equivalents. nd: not detected. tr: traces. Data are given as mean ± standard deviation ($n = 3$). Mean values within a line with different letters are significantly different (homogenous groups) at $p \leq 0.05$.

Table S3. Pearson's correlation coefficients and significance level.

	PT	CUPRAC	FRAP	DPPH$^{•}$	ABTS$^{•+}$
CUPRAC	0.9808***				
FRAP	0.9740***	0.9653***			
DPPH$^{•}$	0.9579***	0.9454***	0.9747***		
ABTS$^{•+}$	0.9850***	0.9799***	0.9787***	0.9643***	
Total flavonoids	0.1921	0.1928	0.2349	0.2138	0.1744
Total hydroxycinnamic acids	0.3015*	0.3311*	0.3066*	0.2979*	0.2862*
Total hydroxybenzoic acids	0.5485***	0.5709***	0.4830**	0.4746**	0.5342***
Total polyphenols (HPLC)	0.2741	0.2829	0.2977*	0.2791	0.2561
Total compounds (HPLC)	0.1243	0.1546	0.1198	0.1113	0.1003
Luteolin 7-*O*-rutinoside	0.1952	0.2182	0.2266	0.2013	0.1689
Luteolin 7-*O*-glucoside	0.1694	0.1776	0.1829	0.1677	0.1367
Apigenin rutinoside	0.1089	0.1218	0.1831	0.1785	0.0996
Apigenin glucuronide	0.1668	0.1583	0.2098	0.1619	0.1599
Luteolin acetyl-glucoside	-0.2084	-0.1899	-0.1131	-0.1279	-0.1769
Luteolin	0.5362***	0.5179***	0.5486***	0.5162***	0.5451***
Apigenin	0.3769**	0.3393*	0.3373*	0.2853*	0.3461*
Other flavonoids	-0.2894*	-0.3045*	-0.1996	-0.1773	-0.2864*
Chlorogenic acid	0.2570	0.2869*	0.2595	0.2577	0.2338
Coumaroyl-quinic acid	0.2191	0.2487	0.2371	0.2473	0.2014
Di-caffeoylquinic acid I	0.3537*	0.3398*	0.2125	0.2692	0.2857*
Di-caffeoylquinic acid II	0.0483	0.0408	0.0187	0.0016	0.0265
Other hydroxycinnamic acids	0.3779**	0.3997**	0.4037**	0.3610*	0.4043**
Protocatechuic acid hexoside	0.5027***	0.5848***	0.4460***	0.4194**	0.4716***
Other hydroxybenzoic acids	0.4743***	0.4685***	0.4166**	0.4171**	0.4695***
Tryptophan	-0.2148	-0.2516	-0.2115	-0.2084	-0.2314
Cynaropicrin	-0.1641	-0.1204	-0.1622	-0.1699	-0.1812
Pinoresinol hexoside	0.6111***	0.5907***	0.5263***	0.5082***	0.5637***

* significant at $p \leqslant 0.05$; ** significant at $p \leqslant 0.01$; *** significant at $p \leqslant 0.001$

Table S4. Regression coefficient of polynomial function of the most significant response surfaces during UAE

Term	Coefficients	Standard Error	*F*-Value	*p*-Value
luteolin 7-O-rutinoside				
Intercept	15.43	1.50		
X_1	1.17	1.18	0.9770	0.3559
X_2	0.3038	1.18	0.0658	0.8049
X_3	3.14	1.18	7.05	0.0327
X_1X_2	-0.8450	1.67	0.2548	0.6292
X_1X_3	1.60	1.67	0.9192	0.3696
X_2X_3	1.12	1.67	0.4456	0.5258
X_1^2	8.01	1.63	24.10	0.0017
X_2^2	-1.99	1.63	1.48	0.2627
X_3^2	3.07	1.63	3.55	0.1017
luteolin 7-O-glucoside				
Intercept		1.41		
X_1	3.84	1.11	11.90	0.0107
X_2	1.41	1.11	1.59	0.2471
X_3	5.08	1.11	20.81	0.0026
X_1X_2	-0.4600	1.57	0.0855	0.7785
X_1X_3	2.84	1.57	3.26	0.1138
X_2X_3	-1.33	1.57	0.7090	0.4276
X_1^2	0.6002	1.53	0.1532	0.7072
X_2^2	-3.82	1.53	6.21	0.0415
X_3^2	1.90	1.53	1.54	0.2553
apigenin rutinoside				
Intercept	6.56	1.27		
X_1	1.83	1.00	3.35	0.1100
X_2	-0.7175	1.00	0.5140	0.4966
X_3	-1.78	1.00	3.16	0.1188
X_1X_2	-1.49	1.42	1.10	0.3282
X_1X_3	-3.55	1.42	6.29	0.0405
X_2X_3	0.0875	1.42	0.0038	0.9524
X_1^2	4.50	1.38	10.66	0.0138
X_2^2	7.72	1.38	31.29	0.0008
X_3^2	-7.51	1.38	29.65	0.0010
luteolin				
Intercept	9.10	1.62	63.53	< 0.0001
X_1	-10.21	1.28	0.7753	0.4078
X_2	1.13	1.28	0.5120	0.4974
X_3	-0.9163	1.28	5.21	0.0564
X_1X_2	4.13	1.81	11.04	0.0127

X_1X_3	-6.02	1.81	0.4880	0.5074
X_2X_3	-1.26	1.81	25.95	0.0014
X_1^2	8.99	1.77	2.40	0.1650
X_2^2	-2.74	1.77	0.9014	0.3740
X_3^2	1.68	1.77	63.53	< 0.0001
chlorogenic acid				
Intercept	47.74	2.32		
X_1	28.32	1.83	238.43	< 0.0001
X_2	-1.63	1.83	0.7852	0.4050
X_3	0.1850	1.83	0.0102	0.9225
X_1X_2	2.36	2.59	0.8316	0.3921
X_1X_3	-1.03	2.59	0.1562	0.7044
X_2X_3	1.33	2.59	0.2630	0.6239
X_1^2	-16.26	2.53	41.36	0.0004
X_2^2	2.10	2.53	0.6918	0.4330
X_3^2	-1.26	2.53	0.2475	0.6341
cynaropicrin				
Intercept	74.52	1.61	1447.20	< 0.0001
X_1	48.39	1.27	0.6862	0.4348
X_2	1.05	1.27	0.7874	0.4044
X_3	-1.13	1.27	0.0095	0.9252
X_1X_2	-0.1750	1.80	0.1150	0.7445
X_1X_3	-0.6100	1.80	4.73	0.0661
X_2X_3	3.91	1.80	267.19	< 0.0001
X_1^2	-28.66	1.75	2.74	0.1420
X_2^2	2.90	1.75	1.54	0.2546
X_3^2	2.18	1.75	1447.20	< 0.0001

Table S5. Analysis of variance (ANOVA) of the selected modelled responses during UAE.

Source	Sum of Squares	Degree of Freedom	Mean Square	*F*-Value	*p*-Value
luteolin 7-O-rutinoside					
The recovery					
Model	438.60	9	48.73	4.35	0.0328
Residual	78.47	7	11.21		
Lack of fit	74.84	3	24.95	27.52	0.0039
Pure error	3.63	4	0.9066		
Total	517.06	16			
$R^2 = 0.8482$					
luteolin 7-O-glucoside					
The recovery					
Model	454.75	9	50.53	5.10	0.0215
Residual	69.33	7	9.90		
Lack of fit	65.20	3	21.73	21.04	0.0065
Pure error	4.13	4	1.03		
Total	524.08	16			
$R^2 = 0.8677$					
apigenin rutinoside					
The recovery					
Model	665.73	9	73.97	9.23	0.0039
Residual	56.09	7	8.01		
Lack of fit	55.88	3	18.63	350.63	< 0.0001
Pure error	0.2125	4	0.0531		
Total	721.82	16			
$R^2 = 9223$					
luteolin					
The recovery					
Model	1449.18	9	161.02	12.28	0.0016
Residual	91.82	7	13.12		
Lack of fit	83.66	3	27.89	13.66	0.0144
Pure error	8.17	4	2.04		
Total	1541.01	16			
$R^2 = 0.9404$					
chlorogenic acid					
The recovery					
Model	7607.22	9	845.25	31.42	< 0.0001
Residual	188.33	7	26.90		
Lack of fit	121.98	3	40.66	2.45	0.2033
Pure error	66.36	4	16.59		
Total	7795.56	16			

$R^2 = 0.9758$					
	cynaropicrin				
The recovery					
Model	22286.05	9	2476.23	191.28	< 0.0001
Residual	90.62	7	12.95		
Lack of fit	15.28	3	5.09	0.2705	0.8444
Pure error	75.34	4	18.83		
Total	22376.67	16			
$R^2 = 0.9960$					

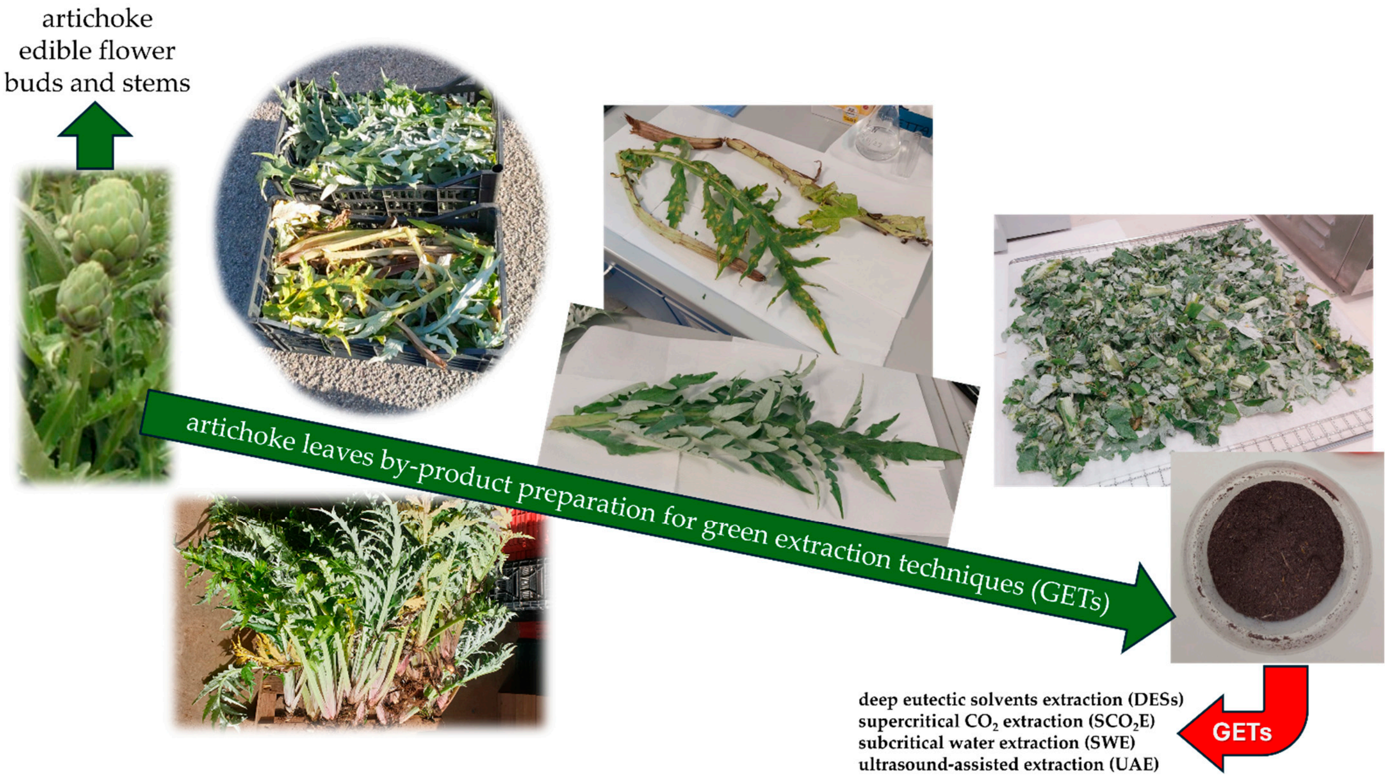

Figure S1. Separation and preparation of C. *cardunculus* var. *scolymus* leaves by-products from edible portion (flower buds and stems).

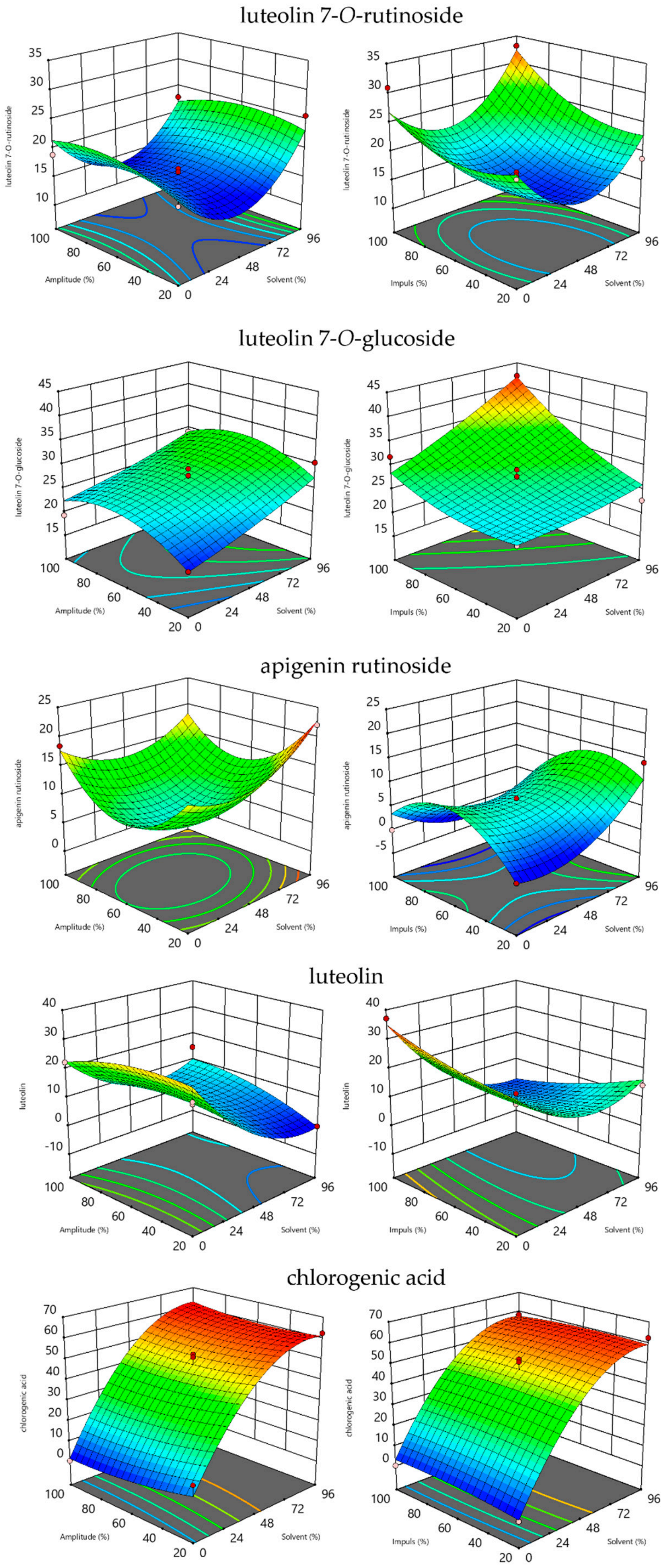

Figure S2. Three-dimensional plots for obtained compounds (luteolin 7-*O*-rutinoside, luteolin 7-*O*-glucoside, apigenin rutinoside, luteolin, chlorogenic acid) in extracts as a function of UAE process parameters.
